# Cognitive implications and associated transcriptomic signatures of distinct regional iron depositions in cerebral small vessel disease

**DOI:** 10.1002/alz.70196

**Published:** 2025-04-21

**Authors:** Youjie Wang, Chen Ye, Ruosu Pan, Biqiu Tang, Congjun Li, Junfeng Liu, Wendan Tao, Xuening Zhang, Tang Yang, Yuying Yan, Shuai Jiang, Su Lui, Bo Wu

**Affiliations:** ^1^ Department of Neurology West China Hospital of Sichuan University Chengdu China; ^2^ Center of Cerebrovascular Diseases West China Hospital of Sichuan University Chengdu China; ^3^ Department of Radiology West China Hospital of Sichuan University Chengdu China

**Keywords:** Allen Human Brain Atlas, cerebral small vessel disease, gene expression, iron deposition, quantitative susceptibility mapping

## Abstract

**INTRODUCTION:**

Regional brain iron dyshomeostasis is observed in cerebral small vessel disease (cSVD) and other neurodegeneration processes. However, its spatial patterns, cognitive impact, and underlying pathological mechanisms remain unclear.

**METHODS:**

Voxel‐based analysis of quantitative susceptibility mapping (QSM) was used to detect regional susceptibility changes, and their correlations with cognitive function were assessed using linear regression. We combined the microarray dataset from the Allen Human Brain Atlas (AHBA) to explore the pathological mechanisms of iron deposition patterns.

**RESULTS:**

A total of 87 cSVD patients and 80 controls were included in the study. Increased QSM values in the bilateral putamen and caudate were associated with cognitive decline in cSVD. Gene set enrichment analysis revealed the enrichment of gene sets related to central nervous system integrity.

**DISCUSSION:**

Iron deposition in deep gray matter may indicate cognitive changes in cSVD and could be linked to the disruption of brain structural and functional integrity.

**Highlights:**

- Increased susceptibility values, indicating focal iron deposition, were observed in the deep gray matter of patients with cerebral small vessel disease (cSVD).
- Regional iron concentration in the deep gray nuclei was associated with cognitive impairment in cSVD patients.
- Imaging transcriptomics suggests that cSVD‐related iron deposition is linked to the structural and functional integrity of the brain.
- An open‐source script for imaging transcriptomics focusing on regional gene expression was developed and proposed.

## 1 BACKGROUND

Cerebral small vessel disease (cSVD) is a common chronic brain vascular disease that affects the small arteries, arterioles, capillaries, and veins through a variety of pathological processes. Several magnetic resonance imaging (MRI) markers, such as white matter hyperintensities (WMHs), perivascular spaces (PVSs), and cerebral microbleeds (CMBs), have been widely used to characterize cSVD patients. These neuroimaging features have also been found to be associated with lacunar strokes and vascular cognitive impairment caused by cSVD, though the underlying mechanisms remain unclear.^1^ Beyond these imaging markers reflecting vascular dysfunction, the role of iron deposition in cSVD has gained significant attention as iron is involved in many critical biological processes such as oxidative phosphorylation, myelination, and the metabolism of neurotransmitters in the central nervous system (CNS).^2^ Previous research on cerebral autosomal dominant arteriopathy with subcortical infarcts and leukoencephalopathy (CADASIL), a hereditary cSVD, supported the relationship between iron accumulation with disease burden and cognitive impairment.^3^ Focal iron deposition in the globus pallidus was proposed as an imaging maker for cerebral vascular degeneration in cSVD patients.^4^ The synergistic effect of focal iron changes and cSVD burden on cognitive function has also been explored.^5^

Compared to conventional T2*‐weighted imaging or susceptibility‐weighted imaging (SWI), quantitative susceptibility mapping (QSM) offers a more advanced approach by providing quantitative information about the magnetism of brain tissue through the reconstruction of a susceptibility map (also known as the χ map). As a non‐invasive method for brain mineral content measurement, QSM has been applied to identify abnormal iron metabolism and has shown utility in reflecting disease progression across various neurological diseases, including Alzheimer's disease (AD), Parkinson's disease (PD), multiple sclerosis (MS), and Friedreich's ataxia.^2^ Concerning the cSVD population, studies have reported that QSM‐derived iron deposition is associated with cerebral vascular degeneration and cognitive decline.^3^, ^4^

To better understand the derivation of the brain QSM signal and explore its clinical application, genome‐wide association studies (GWASs) and exome‐wide association studies (EWASs) have been conducted to identify genes that are significantly linked with brain iron.^6^, ^7^ In addition to genes related to iron transport and homeostasis, several biological functions involving calcium, extracellular matrix, and myelin also contributed to the QSM‐derived susceptibility map. To overcome the weakness of using expression data from blood samples rather than brain tissue in these studies, the Allen Human Brain Atlas (AHBA) datasets were used to characterize the relationship between QSM signal, especially in the subcortical nucleus, and genes.^8^, ^9^ However, studies combining susceptibility maps specifically in the cSVD population and brain gene expression data are still lacking.

On this basis, we aimed to answer the following scientific questions in the present study: (1) Is there any difference in the susceptibility value between cSVD patients and healthy controls in specific brain regions? (2) Is the susceptibility value in specific regions related to cognitive function in the study population, and does the severity of cSVD interact with the degree of iron deposition on cognitive performance? (3) Could the spatial pattern of susceptibility differences be linked to the spatial distribution of specific functional gene pathways?

## 2 METHODS

### 2.1 Study population

**FIGURE 1 alz70196-fig-0001:**
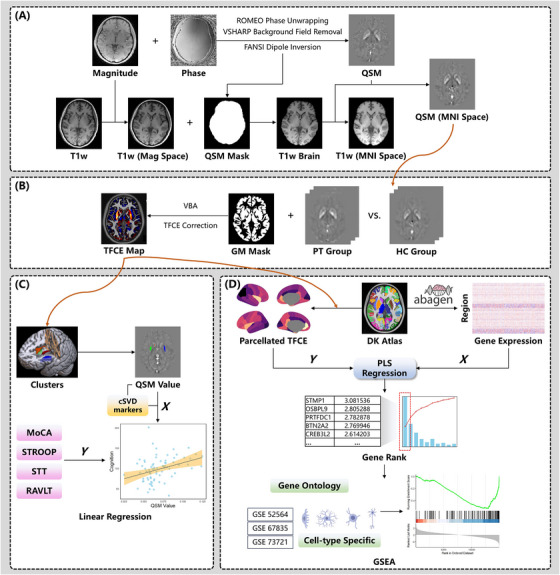
Schematic summary of the study design. A, QSM preprocessing and reconstruction. The magnitude and phase imaging were used to generate the susceptibility map in native space. The QSM imaging was then normalized to the MNI space using the affine parameters derived from registering T1‐weighted imaging in the space of the first echo magnitude imaging to the standard MNI template. B, Voxel‐wise QSM analysis. We compared the QSM value in gray matter between cSVD patients and healthy controls, resulting in a TFCE map. C, Correlation between regional QSM value and cognitive function. We extracted the mean susceptibility value in the statistically significant clusters and explored the relationship between iron deposition and cognitive function. D, Imaging transcriptomics analysis. In the main analysis, the volumetric DK atlas was used to generate a regional TFCE map and gene expression matrix. We performed PLS regression to acquire ranked gene lists representing their correlation with the parcellated TFCE value, which was consequently used to conduct the GSEA, seeking enriched gene sets, to explain the iron deposition pattern in cSVD. cSVD, cerebral small vessel disease; DK, Desikan–Killiany; FANSI, fast non‐linear susceptibility inversion; GM, gray matter; GSEA, gene set enrichment analysis; HC, healthy controls; MNI, Montreal Neurological Institute; MoCA, Montreal Cognitive Assessment; PLS, partial least squares; PT, patients; QSM, quantitative susceptibility mapping; RAVLT, Rey Auditory Verbal Learning Test; ROMEO, rapid opensource minimum spanning tree algorithm; STROOP, Stroop Color and Word Test; STT, Shape Trail Test; TFCE, threshold‐free cluster enhancement; VBA, voxel‐based analysis; VSHARP, variable kernel sophisticated harmonic artifact reduction for phase data.

Figure 1 provides a schematic overview of the study design in this research. We prospectively and consecutively collected cSVD patients admitted to West China Hospital from July 2021 to December 2023. This study was approved by the biomedical research ethics committee of West China Hospital, Sichuan University (No. 2020[922]) and followed the tenets of the Declaration of Helsinki. Patients were eligible if they had symptoms consistent with cSVD (either a clinical lacunar stroke with an anatomically corresponding lacunar infarct, gait apraxia, or self‐reported cognitive impairment), and moderate or higher WMH burden on MRI scan (Fazekas score ≥ 2). The exclusion criteria were: (1) previous history of stroke or other neurological disorder; (2) evidence of cardioembolism confirmed by electrocardiograph or echocardiography, monogenic forms of cSVD, cerebral amyloid angiopathy, and any other cause of stroke other than cSVD; (3) contraindications to MRI. Age‐ and sex‐matched healthy controls were recruited via advertisement from native communities in Chengdu city during the same period. All participants underwent a battery of neuropsychological tests including the following: Mini‐Mental State Examination (MMSE) and Montreal Cognitive Assessment‐Beijing version (MoCA) for global cognition, Chinese Rey Auditory Verbal Learning Test (C‐RAVLT) for immediate and delayed verbal memory, and the Stroop Color and Word Test (SCWT) with Shape Trail Test (STT) for executive function.

### 2.2 MRI protocols and cSVD imaging markers assessment

**FIGURE 2 alz70196-fig-0002:**
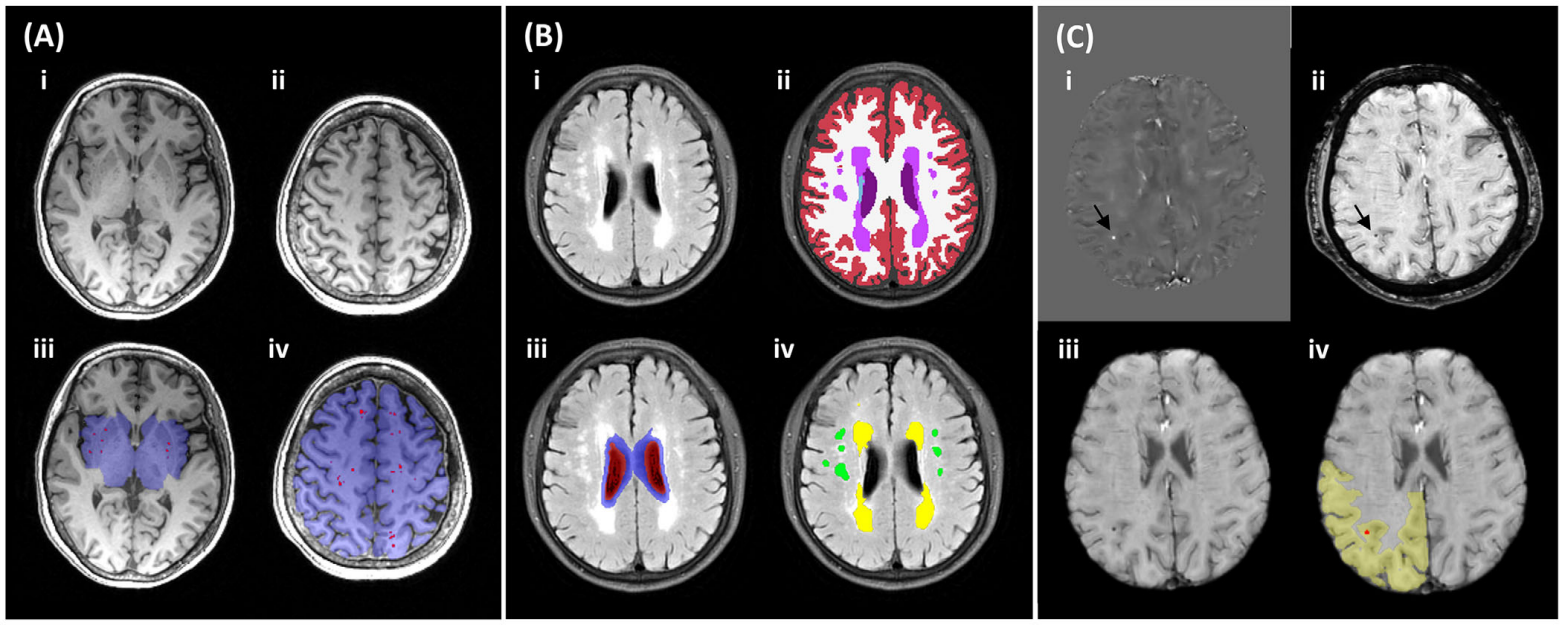
The quantification of cSVD imaging markers. Ai and Aii, The subject's T1w images. Aiii and Aiv, The corresponding PVS segmentation results (in red) along with the basal ganglia and deep white matter region masks (in transparent blue), which were obtained using the SHiVAi pipeline. Bi, Representative axial slice from a T2 FLAIR image. Bii, Corresponding WMH segmentation result obtained using WMH‐SynthSeg. PWMH (Biv, marked in yellow) and DWMH (Biv, marked in green) were distinguished based on whether any voxel of a given WMH cluster was located within 3 mm of the lateral ventricle (Biii: the lateral ventricles are highlighted in transparent red, and the periventricular mask within 3 mm is shown in transparent blue). Ci and Cii, A representative CMB on QSM and SWI images, respectively. Ciii, Skull‐stripped SWI image, registered to the T1w image, which serves as the input for the SHIVA‐CMB model. Civ, Detected CMB lesion (in red), classified as a lobar CMB because it falls within the right parietal region (in transparent yellow) given by SHiVAi. CMB, cerebral microbleed; DWMH, deep white matter hyperintensity; FLAIR, fluid‐attenuated inversion recovery; PVS, perivascular space; PWMH, periventricular WMH; QSM, quantitative susceptibility mapping; SWI, susceptibility‐weighted imaging; WMH, white matter hyperintensity.

All enrolled subjects underwent MRI scanning on a 3.0 T scanner (SIGNA Premier, GE Medical Systems) with a 48‐channel head coil. Imaging data were collected on the same day that the cognitive tests were completed. The detailed acquisition protocol has been described previously.^10^, ^11^ The typical MRI markers, including lacunes, WMHs, PVSs, and CMBs, were rated according to the Standards for Reporting Vascular Changes on Neuroimaging (STRIVE) consensus criteria.^12^ The specific visual assessment process has been elaborated elsewhere.^13^ We also quantified the extent of WMHs, PVSs, and CMBs using automated methods. The FreeSurfer WMH‐SynthSeg^14^ was used to segment WMHs based on axial T2 fluid‐attenuated inversion recovery (FLAIR) images, as it provides robust results on low‐resolution MRI images. The results were further divided into periventricular WMHs (PWMHs) and deep WMHs (DWMHs), with the classification based on whether any voxel within a WMH cluster was located within 3 mm of the lateral ventricles.^15^ PVSs and CMBs were segmented using the SHiVAi pipeline (https://github.com/pboutinaud/SHiVAi),^16^, ^17^ and classified according to the brain regions identified by FreeSurfer SynthSeg.^18^ PVS clusters segmented from T1‐weighted images were categorized as basal ganglia PVSs (BG PVSs) and deep white matter PVSs (WM PVSs).^19^ SWI images derived from CLEAR‐SWI^20^ using phase and magnitude images in QSM sequences were used for CMB segmentation. The segmentation results were reviewed by an experienced neurologist (C.Y.) to avoid misidentifications, such as vessels, calcification, and air‐bone interfaces. According to the Microbleed Anatomical Rating Scale (MARS),^21^ lobar CMBs were determined based on the presence of CMBs in the frontal, temporal, occipital, and parietal lobes. Figure 2 illustrates the quantitative process of cSVD imaging markers. The volume data of PVSs and WMHs were expressed as a proportion of intracranial volume (%ICV) to account for differences in brain volume across subjects.

**RESEARCH IN CONTEXT**

1. **Systematic review**: The authors reviewed the literature using traditional sources. While consistent evidence from animal and clinical studies supports the association between brain iron deposition and cognitive decline in various neurological diseases, a deeper understanding of the specific iron concentration pattern and possible pathological mechanisms is needed to fundamentally understand cerebral small vessel disease (cSVD) pathology. These relevant citations are appropriately referenced.
2. **Interpretation**: Our findings led to an integrated hypothesis explaining the role of common cerebrovascular pathology in region‐selective iron deposition in cSVD patients. This hypothesis is consistent with previous findings concerning different neurological disorders.
3. **Future directions**: Acknowledging the limitations of linking in vivo imaging data with gene expression data derived from *post mortem* tissue, there is a need to develop comprehensive models that can directly connect gene expression in brain tissue with neuroimaging data. Longitudinal studies are also warranted to investigate the role of iron deposition in long‐term cognitive changes.

### 2.3 QSM preprocessing and voxel‐based analysis

The QSM maps were reconstructed from the raw multi‐echo phase and magnitude data using the SEPIA toolbox (version 1.2.2.6, https://github.com/kschan0214/sepia). The QSM reconstruction pipeline followed the[Fig alz70196-fig-0001] recommendation[Fig alz70196-fig-0002] from a recently published consensus for clinical QSM research.^22^ After correcting the inter‐slice opposite polarity on real and imaginary images, the phase image was inverted before the main QSM reconstruction processing, so that the paramagnetic susceptibility was present as a positive value in the final QSM maps. Specifically, we performed phase unwrapping using the rapid opensource minimum spanning tree algorithm (ROMEO) along with MCPC‐3D‐S coil combination to remove the phase offsets and recover the true phase data.^23^, ^24^ Then, the background field contribution was removed with variable kernel sophisticated harmonic artifact reduction for phase data (VSHARP) methods.^25^ The final susceptibility maps were reconstructed using the fast non‐linear susceptibility inversion (FANSI) algorithm.^26^ To obtain QSM maps in standard Montreal Neurological Institute (MNI) space, the T1‐weighted structural image was registered to the magnitude image of the first echo. The registered T1‐weighted image was skull‐stripped with the mask used for QSM reconstruction and normalized to MNI space using FSL (version 6.0, https://fsl.fmrib.ox.ac.uk/fsl) and ANTs (version 2.5.1, https://github.com/ANTsX/ANTs). Therefore, the QSM maps could be transformed into MNI space with the affine parameters generated in the former steps.

QSM images in MNI space (voxel size: 1 × 1 × 1 mm^3^) were smoothed with a 3 mm full width at half maximum Gaussian kernel taken from previous voxel‐based QSM studies.^27^ Second‐level analysis of QSM maps was conducted with statistical parametric mapping (SPM12, https://www.fil.ion.ucl.ac.uk/spm/) software. Age and sex were set as the covariates. We excluded WM from the whole‐brain analysis by using a gray matter mask derived from the MNI152 1 mm‐resolution template to reduce the interference from myelin density and the orientation of WM fibers, as described in previous research.^3^ The threshold‐free cluster enhancement (TFCE) with 5000 permutations was adopted to correct for multiple comparisons, using the TFCE toolbox (version r269, http://dbm.neuro.uni‐jena.de/tfce). We established a family‐wise error (FWE) corrected threshold of *P* < 0.05. A voxel‐based morphometry (VBM) analysis with age, sex, and total intracranial volume (TIV) as covariates was also conducted to rule out the effect of gray matter volume on the detected susceptibility difference in the statistically significant clusters, with the same significance level.

### 2.4 Statistical analysis

Demographics and clinical characteristics were presented as mean ± standard deviation (SD) for continuous variables, median with interquartile ranges for count variables, and frequencies with percentages for categorical variables. Between‐group differences in baseline characteristics were assessed by the Wilcoxon rank‐sum test and Pearson chi‐squared test. Age‐ and sex‐adjusted partial correlation analysis was used to describe the relationship between regional susceptibility values and cSVD markers. Generalized linear model (GLM) analyses were conducted to explore the relationship between QSM values in specific clusters and cognitive performance with age, sex, education level, and other cerebrovascular risk factors including hypertension, hyperlipidemia, diabetes mellitus, and smoking as confounders (Model I). In the second model, we additionally adjusted for total WMH volume, total PVS volume, and lobar CMB (Model II). The statistical threshold was set to a *P* value of 0.05. To investigate whether cSVD markers moderate the relationship between iron deposition and cognitive function, we included interaction terms for QSM value × cSVD markers alongside the main effect terms in the model. The data were scaled, and the same confounders as in Model I were adjusted for. Because we explored the interactions of eight different cSVD‐related markers, the significance threshold for the interactions was set to 0.00625 (0.05/8) for multiple comparisons. All statistical analyses mentioned in this section were conducted with R version 4.4.1.

### 2.5 Imaging transcriptomics analysis

The overall framework of neuroimaging transcriptomics analysis in the present study followed the guidelines provided by Arnatkeviciute et al.^28^, ^29^ The idea of linking statistical maps or other neuroimaging phenotypes with gene expression measured in *post mortem* brains has been verified by studies referring to various brain disorders.^30^, ^31^ To ensure the transparency of this study, the entire imaging transcriptomics analysis process can be reproduced using the code available at https://github.com/LuuuXG/Brain‐Imaging‐Transcriptomics‐Scripts.

Regional microarray expression data were obtained from six *post mortem* brains (1 female, ages 24.0–57.0, 42.50 ± 13.38) provided by the AHBA (https://human.brain‐map.org).^32^ Data were processed with the abagen toolbox (version 0.1.3; https://github.com/rmarkello/abagen) using the Desikan–Killiany volumetric atlas with 83 regions in MNI space.^33^ Two regions (right frontal pole and temporal pole) were not matched to any tissue samples because only two of the six donors had tissue samples taken from the right hemisphere. The expression values in these two regions were interpolated by assigning every node in the region to the expression of the nearest tissue sample to generate a dense expression matrix with 83 rows (regions) and 15,633 columns (genes). The detailed processing steps can be found in the supporting information.

Partial least squares (PLS) methods were adopted to identify genes significantly associated with the QSM value differences between cSVD and control group.^34^ The whole brain non‐parametric TFCE statistic map generated from voxel‐based analysis (VBA) was parcellated with the Desikan–Killiany atlas to represent the region of interest (ROI)‐level group difference. Meanwhile, correspondence in our analysis in the volumetric MNI 152 space, the ROI‐level spatial nulls (*n* = 1000), which maintained the spatial autocorrelation properties of the parcellated data, were simulated for the subsequent genes ranking with the neuromaps toolbox (version 0.0.5, https://github.com/netneurolab/neuromaps).^35^ We conducted PLS regression (PLSR), which has been applied in brain imaging transcriptomics analysis,^30^, ^36^ to calculate the weights of each independent variable (i.e., 15,633 genes) in each PLS component. Both the regional gene expression matrix and regional TFCE map were *z* score transformed beforehand so that the weights of genes could represent the directional (i.e., both positive and negative) contribution of a certain gene to a PLS component. We used bootstrap methods (*n* = 1000) to quantify the directional association between each gene and the TFCE map in each component. Specifically, the weights of genes were first taken to the opposite number if the PLS component scores positively correlated with the TFCE map to ensure the weights could reflect the directional correlation with the TFCE map. Then, a *Z* statistic value for each gene, defined by the modified weight divided by the SD of modified weights generated from PLSR using spatial nulls, was calculated and used for gene importance ranking.

To obtain gene sets referring to specific cell types, we used the RNA‐seq data from three previous studies (GSE52564,^37^ GSE67835,^38^ and GSE73721^39^), which have been used in similar human brain imaging transcriptomics research.^36^, ^40^ For each series, the fragments per kilobase of transcript per million mapped reads (FPKM) values were fetched from the Gene Expression Omnibus (GEO) repository and averaged across samples for endothelial cells, neurons, oligodendrocytes, astrocytes, microglia, and oligodendrocyte precursor cells (OPCs). Specificity index (SI) analysis was applied to determine genes specific for each cell type using the pSI R package (version 1.1, https://sites.wustl.edu/doughertylab/psi_package‐page/).^41^ Specifically, the SI of each gene was calculated as the average rank across comparisons between the FPKM of different cell types. Then, a *P* value for SI (pSI) was estimated through permutation testing. The threshold of 0.001 was taken to define the cell type–specific gene list in each of the three series.

Gene set enrichment analysis (GSEA), a data‐driven analysis test, was consequently used for exploring whether certain sets of functionally related genes were strongly associated with the gene‐phenotype obtained from PLSR analysis. To facilitate the interpretation of the results, we performed GSEA using the gene ranking from only the first PLSR component, as it explained the most variance between gene expression and TFCE values. GSEA was performed with the “gseGO” function for Gene Ontology (GO) terms and with the “GSEA” function for cell type–specific gene sets in the clusterProfiler R package.^42^ Normalized enrichment score (NES) and false discovery rate (FDR) were used to quantify the enrichment magnitude and statistical significance, respectively.

## 3 RESULTS

### 3.1 Baseline characteristics

Baseline characteristics, including demographic data, cerebrovascular risk factors, cognitive performance, and imaging measures of the patients with cSVD and healthy controls are listed in Table 1. Overall, 167 individuals were included in the present study. There were no significant differences between patients and healthy controls in age (*P* = 0.500), sex (*P* = 0.090), and education (*P* = 0.800). Patients with cSVD showed more vascular risk factors. Impaired cognitive function was found in the different cognitive domains, including global cognition (MoCA), executive function (SCWT), and memory (C‐RAVLT immediate recall and delayed recall).

**TABLE 1:** Baseline characteristics of the study population.

	cSVD (*n* = 87)	Controls (*n* = 80)	*P* value
**Demographics**			
Age (years)	59.29 (10.93)	58.96 (8.05)	0.5
Male (male/female)	62/25	47/33	0.090
Education (years)	10.59 (4.63)	10.85 (4.18)	0.8
**Cerebrovascular risk factors**			
Smoke	42 (48%)	14 (18%)	<0.001
Alcohol	34 (39%)	16 (20%)	0.007
Hypertension	47 (54%)	15 (19%)	<0.001
Diabetes	29 (33%)	5 (6.3%)	<0.001
Hyperlipidemia	13 (15%)	4 (5.0%)	0.034
**Imaging features**			
TIV	1469.86 (125.30)	1476.61 (127.22)	0.8
GM	633.63 (54.07)	636.54 (48.98)	0.7
WM	497.94 (64.46)	498.58 (56.60)	0.8
***Visual scoring***			
Lacune	15 (17%)	5 (6.3%)	0.029
PWMH	1 (0–1)	0 (0–1)	0.014
DWMH	0 (0–1)	0 (0–)	0.3
BG‐PVS	1 (0–2)	0 (0–1)	0.023
CSO‐PVS	1 (1–2)	1 (1–1)	0.007
CMB	18 (21%)	4 (5.0%)	0.003
***Quantitative measurements***			
Total PVS volume (%ICV)	0.11 (0.07)	0.11 (0.06)	0.9
BG PVS volume (%ICV)	0.02 (0.01)	0.02 (0.01)	0.3
WM PVS volume (%ICV)	0.09 (0.07)	0.08 (0.06)	0.8
Total WMH volume (%ICV)	0.60 (0.41)	0.35 (0.15)	<0.001
PWMH volume (%ICV)	0.55 (0.40)	0.33 (0.14)	<0.001
DWMH volume (%ICV)	0.04 (0.05)	0.02 (0.03)	<0.001
Lobar CMB	15 (17%)	2 (2.5%)	0.002
**Cognitive function**			
MoCA	23.0 (19.0–26.0)	25.0 (22.0–27.0)	0.003
STT‐A	73.20 (34.90)	72.83 (38.25)	0.7
STT‐B	181.94 (88.38)	171.50 (62.66)	0.8
Stroop‐A_time_	34.85 (11.01)	31.26 (7.87)	0.021
Stroop‐B_time_	57.78 (18.01)	51.39 (24.51)	0.003
Stroop‐C_time_	100.35 (29.28)	89.55 (27.31)	0.002
C‐RAVLT‐A1‐5	36 (27–42)	37 (31–47)	0.057
C‐RAVLT‐A6	6 (4–8)	8 (6–11)	<0.001
C‐RAVLT‐A7	6 (3–9)	7 (5–11)	0.004

Abbreviations: A1‐5, the sum of five consecutive retrievals of the same words list; A6, retrieval of words without reading them again;

A7, retrieval of the words without reading them again after a 30 minute interval; BG, basal ganglia; CMB, cerebral microbleed; C‐RAVLT, The Chinese Rey Auditory Verbal Learning Test; CSO, centrum semiovale; cSVD, cerebral small vessel disease; DWMH, deep white matter hyperintensity; GM, gray matter; ICV, intracranial volume; MoCA, Montreal Cognitive Assessment‐Beijing version; PVS, perivascular space; PWMH, periventricular white matter hyperintensity; STT, Shape Trail Test; TIV, total intracranial volume; WM, white matter.

### 3.2 Whole‐brain QSM analysis

**FIGURE 3:**
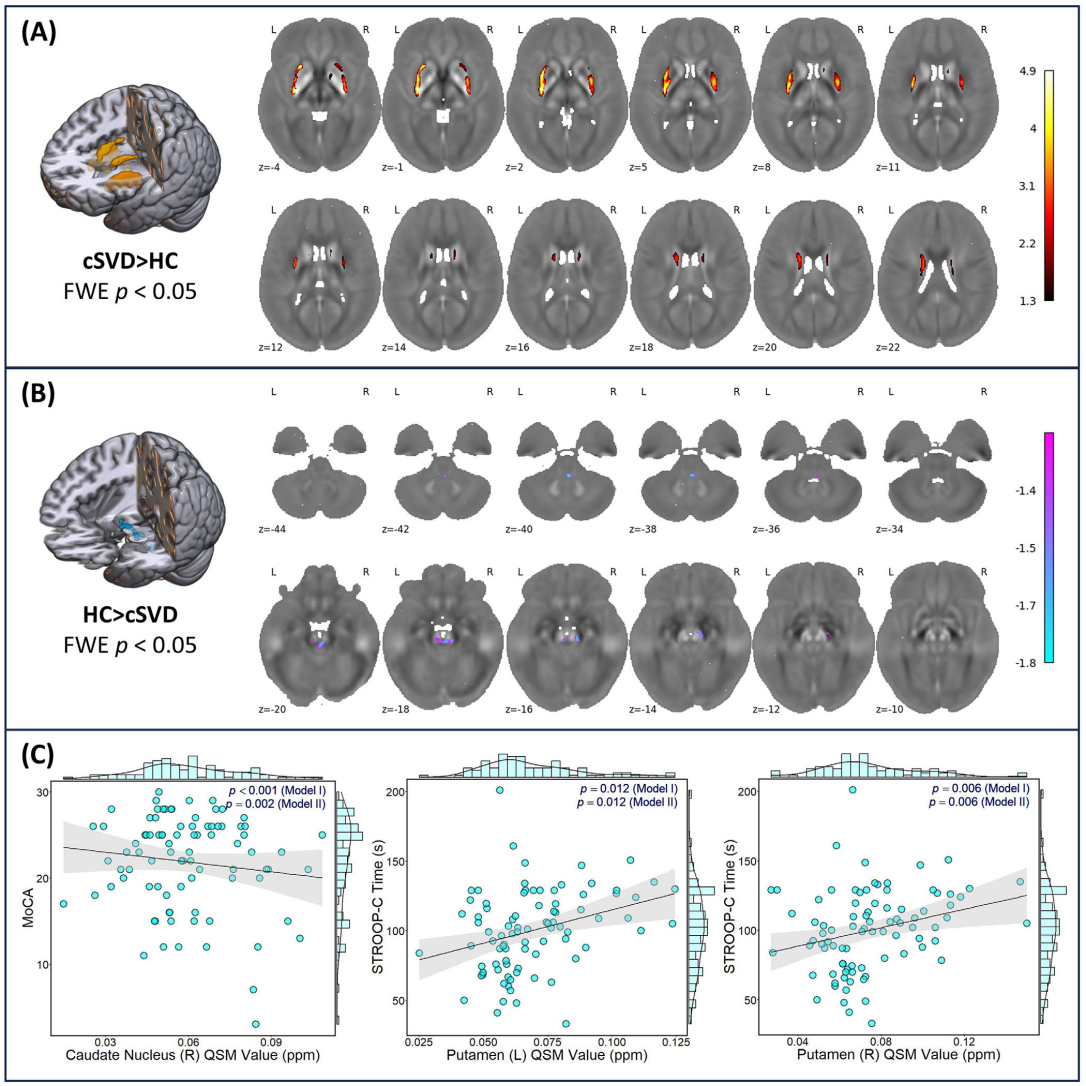
Iron deposition in cSVD and the correlation with cognitive performance. A and B, Results of the voxel‐based analysis revealed focal iron deposition in bilateral putamen and caudate. The log‐transformed *P* values were overlayed on the study‐wise susceptibility maps in the MNI space. C, Scatter plots showed the relationship between increased QSM value and declined cognitive performance in cSVD patients. cSVD, cerebral small vessel disease; FWE, family‐wise error; HC, healthy controls; MNI, Montreal Neurological Institute; MoCA, Montreal Cognitive Assessment; QSM, quantitative susceptibility mapping; STROOP, Stroop Color and Word Test.

Voxel‐wise comparisons of the gray matter QSM value revealed significant iron deposition in the bilateral deep gray matter, especially the putamen and caudate, along with the substantia nigra of the midbrain (Table 2 and Figure 3A). In the healthy control > patient contrast, areas with less pronounced iron deposition in cSVD patients were identified in the brainstem (Table S1 in supporting information and Figure 3B). The result of VBM analysis is shown in Figure S1 in supporting information. These clusters with increased susceptibility were defined as ROIs to extract the mean QSM values for linear regression analysis with cognitive performance in cSVD patients.

**TABLE 2:** Clusters with increased QSM value in cSVD patients compared to controls.

Anatomical structures	Voxel size	Peak TFCE value	*p*‐value (TFCE FWE)	Peak voxel MNI		
				X	Y	Z
Putamen_L	2979	1078.02	<0.001	−29	−10	0
Putamen_R	2326	897.06	<0.001	28	−2	8
Caudate_L	691	742.66	0.001	−16	−7	23
Pallidum_R	184	592.22	0.007	13	3	−6
Midbrain_L^a^	161	543.10	0.013	14	−18	−8
Caudate_R	390	529.02	0.016	15	2	18
Midbrain_R^a^	232	527.42	0.016	−8	−13	−9

*Note*: The anatomical structures were defined according to the Automated Anatomical Labeling (AAL) brain atlas. In subsequent analyses, the clusters were numbered in order of voxel size.

^a^ These two clusters do not correspond to specific anatomical structures in the AAL atlas.

Abbreviations: cSVD, cerebral small vessel disease; FWE, family‐wise error; MNI, Montreal Neurological Institute; QSM, quantitative susceptibility mapping; TFCE, threshold‐free cluster enhancement.

### 3.3 Iron deposition, cSVD imaging markers, and cognitive function

Except for a cluster located in the midbrain, where QSM values were positively correlated with WM PVS volume and PWMH volume, no other regions showed a correlation between iron deposition and cSVD markers (Figure S2 in supporting information). As depicted in Figure 3C, we found the relationship between focal iron deposition and cognitive impairment represented by several cognitive tasks. The susceptibility value in the right caudate was negatively correlated with the MoCA score (*β* = −4.97, 95% confidence interval [CI] = −7.86 to −2.08, *P* < 0.001). Bilateral putamen QSM values showed a consistent positive relation with Stroop‐C_time_ (left putamen: *β* = 421.77, 95% CI = 102.16 to 741.38, *P* = 0.012; right putamen: *β* = 375.18, 95% CI = 114.06 to 636.30, *P* = 0.006). Correlation was also found between QSM value in the right caudate and poorer memory (C‐RAVLT‐A1‐5: *β* = −2.70, 95% CI = −5.00 to −0.40, *P* = 0.022; C‐RAVLT‐A7: *β* = −6.44, 95% CI = −12.13 to −0.75, *P* = 0.026). After adjusting for cSVD imaging markers, the QSM values in the bilateral basal ganglia clusters still showed a relationship with poorer cognitive performance, which was consistent with Model I. Additionally, iron deposition in the bilateral putamen was found to have a statistically significant negative correlation with delayed memory (C‐RAVLT‐A7, left putamen: *β* = −5.51, 95% CI = −10.60 to −0.41, *P* = 0.034; right putamen: *β* = −5.49, 95% CI = −9.87 to −1.11, *P* = 0.014). The complete results of linear regression are demonstrated in Table S2 and S3 in supporting information. Based on the above results, we further investigated whether cSVD status moderated the association between iron deposition in the bilateral basal ganglia clusters and cognitive function. We found a consistent interaction between BG PVS volume and regional QSM values in relation to MoCA scores, with the cluster located in the right putamen surviving multiple comparison corrections (Table 3, Cluster 1 [left putamen] and MoCA: *β* = −0.21, 95% CI = −0.38 to −0.03, *P* for interaction = 0.025; Cluster 2 [right putamen] and MoCA: *β* = −0.29, 95% CI = −0.47 to −0.11, *P* for interaction = 0.003; Cluster 4 [right caudate] and MoCA: *β* = −0.19, 95% CI = −0.34 to −0.04, *P* for interaction = 0.018). The interactions with other cSVD markers can be found in Table S4 in supporting information.

**TABLE 3 alz70196-tbl-0003:** Correlation between QSM value and MoCA with the interaction term QSM value × BG PVS volume.

Predictor	MoCA		
	*β*	95% CI	*P*
**Cluster 1 (left putamen)**	0.05	(−0.31, 0.42)	0.776
BG PVS volume (%ICV)	0.04	(−0.14, 0.21)	0.697
Cluster 1 (left putamen) × BG PVS volume (%ICV)	−0.21	(−0.38, −0.03)	0.025
**Cluster 2 (right putamen)**	0.05	(−0.30, 0.40)	0.780
BG PVS volume (%ICV)	−0.02	(−0.19, 0.16)	0.851
Cluster 2 (right putamen) × BG PVS volume (%ICV)	−0.29	(−0.47, −0.11)	**0.003** ^*^
**Cluster 3 (left caudate)**	0.01	(−0.36, 0.38)	0.967
BG PVS volume (%ICV)	−0.01	(−0.19, 0.16)	0.896
Cluster 3 (left caudate) × BG PVS volume (%ICV)	−0.15	(−0.34, 0.04)	0.123
**Cluster 4 (right caudate)**	−0.14	(−0.49, 0.21)	0.449
BG PVS volume (%ICV)	−0.27	(−0.44, −0.10)	0.003
Cluster 4 (right caudate) × BG PVS volume (%ICV)	−0.19	(−0.34, −0.04)	0.018

*Note*: The data were scaled in the regression models. Age, sex, education level, and vascular risk factors were adjusted.

* *P* value < 0.00625 for the interaction term.

Abbreviations: BG, basal ganglia; CI, confidence interval; ICV, intracranial volume; MoCA, Montreal Cognitive Assessment‐Beijing version; PVS, perivascular space; QSM, quantitative susceptibility mapping; WM, white matter.

### 3.4 Iron deposition pattern and genes

**FIGURE 4 alz70196-fig-0004:**
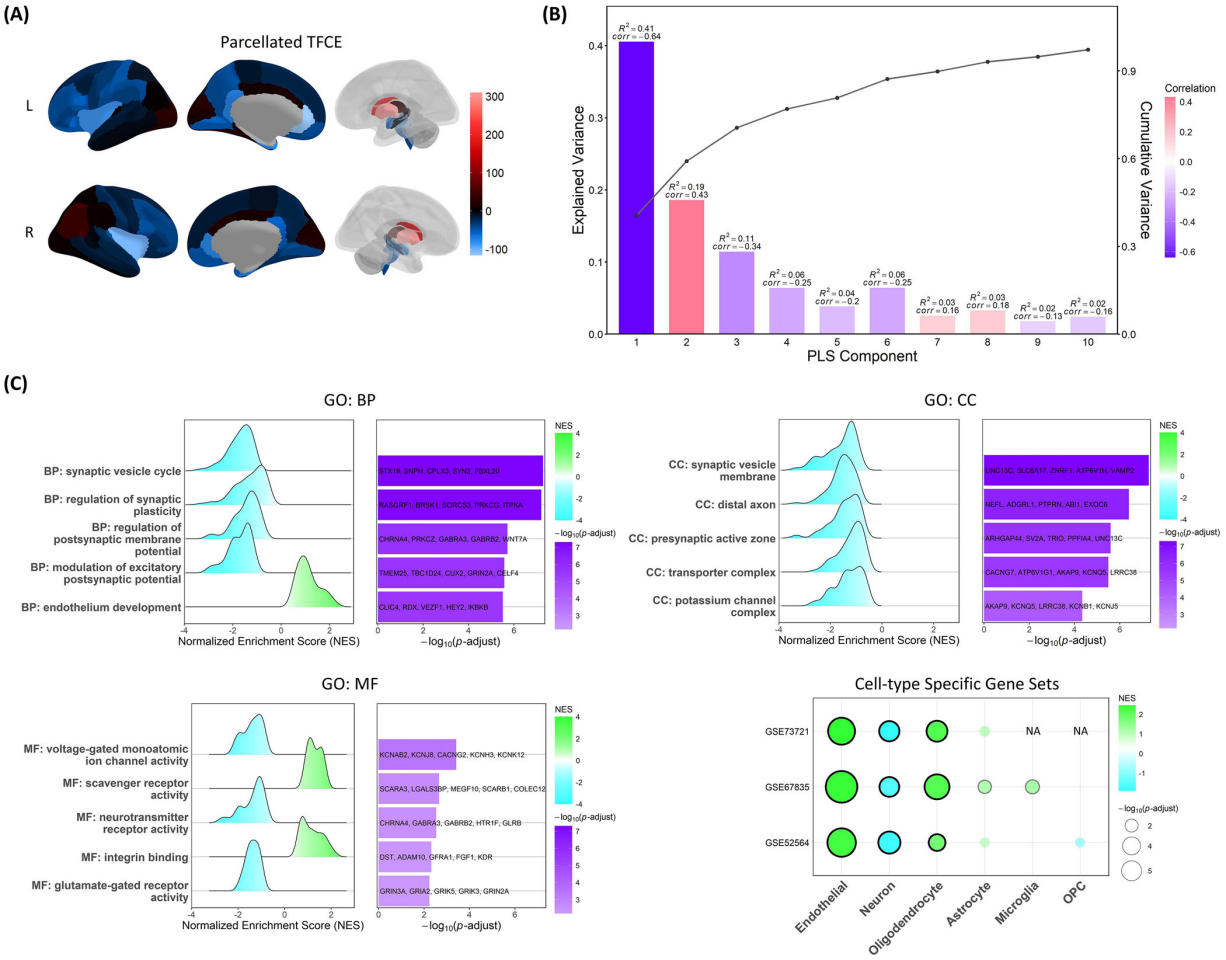
Results of the imaging transcriptomics analysis. A, The parcellated TFCE map. B, The explained variance in the first 10 PLSR components. The correlation coefficient between PLS scores and TFCE value was also annotated and reflected by the bar color. C, Enriched gene sets and their NES, log‐transformed *P* value from GSEA. For GO terms, the top five significant gene sets of BP, CC, and MF were demonstrated with the first five core enrichment gene symbols. Results of cell type–specific gene sets were displayed as the bubble plot. BP, biological process; CC, cellular component; GO, Gene Ontology; GSEA, gene set enrichment analysis; MF, molecular function; OPC, oligodendrocyte precursor cell; PLS, partial least squares; PLSR, partial least squares regression; TFCE, threshold‐free cluster enhancement.

PLSR component 1 (PLSR1) explained 41% of the variance (Figure 4A‐B). There was a negative correlation between TFCE and the scores of PLSR1 (*r* = −0.64). To validate the gene ranking obtained from PLSR, we used a linear regression model to directly correlate the expression of the top 5 and bottom 5 genes from PLSR1 with the parcellated TFCE values. As depicted in Figure S3 in supporting information, the genes ranked at the top in PLSR1 were positively correlated with the TFCE value, while those ranked at the bottom were negatively correlated, consistent with the overall findings from the PLSR analysis.

For GSEA, the enrichment results are displayed in Figure 4C. Several GO terms associated with neuronal signaling and brain structure were enriched in the analysis. The results were simplified to reveal more abundant pathways by calculating the similarity of GO terms and removing those highly similar terms by keeping one representative term. Enrichment for biological process (BP) is involved in endothelium development (GO‐BP: 0003158, normalized enrichment score [NES] = 2.26, –log*P* = 5.51) and several gene sets related to the synapse. With respect to cellular component (CC), in addition to synapse‐related terms enriched at the bottom, the term “distal axon” also showed significant enrichment (GO‐CC: 0150034, NES = –2.07, –log*P* = 6.41). Compared to BP and CC, molecular function (MF) terms showed less statistically significant results, with the term “neurotransmitter receptor activity” enriched at the bottom of the PLSR1 gene list (GO‐MF: 0030594, NES = −1.93, −log*P* = 2.54). The cell type–specific gene sets derived from SI analysis in the present study are demonstrated in supporting information. Gene sets derived from the three different sources revealed generally consistent GSEA results. Gene sets specific to endothelial cells and oligodendrocytes were enriched at the top, while neuron‐specific genes were significantly enriched at the bottom.

### 3.5 Sensitivity analysis

The imaging transcriptomics analysis was repeated (1) using the automated anatomical labeling (AAL) atlas with 90 cerebral regions, and (2) using another transcriptomic dataset, to ensure the robustness of our study. They did not reveal any major discrepancy compared to the main analysis (Figures S4 and S5 in supporting information).

## 4 DISCUSSION

This study compared the QSM‐derived susceptibility maps between cSVD patients and controls at the voxel level. Higher susceptibility values were found in the bilateral putamen and caudate in cSVD patients. In the cSVD group, the susceptibility value in certain deep gray matter areas revealed a negative correlation with cognitive performance. Furthermore, using the GSEA bioinformatics method, we identified several pathways related to brain structure and function that may contribute to the iron deposition patterns observed in cSVD.[Table alz70196-tbl-0003]

We found notable iron deposition in the deep gray matter nuclei, particularly in the bilateral putamen and caudate nucleus, in the cSVD group, as reflected by elevated QSM values. Our analysis did not detect any distinct focal iron concentration in the cortical regions. Additionally, small clusters in the brainstem exhibited lower QSM values, located near the regions of substantia nigra iron deposition, potentially suggesting localized mineral redistribution. Normal iron homeostasis is essential for maintaining the optimal brain function. Disruptions in this balance, clinically assessed using semi‐quantitative or quantitative MRI techniques, have been investigated as potential imaging markers to track disease progression in various neurodegenerative disorders.^2^ Currently, the relationship between brain iron dyshomeostasis and cognitive decline is well established. On the one hand, iron overload can lead to excessive production of hydroxyl radicals, a highly reactive oxygen species (ROS), through the reaction of ferrous iron with hydrogen peroxide, known as the Fenton's reaction, which further results in DNA damage, lipid peroxidation, and mitochondrial dysfunction.^43^ On the other hand, ferroptosis, a recently identified iron‐dependent form of necrosis, is also considered a key mechanism by which iron accumulation contributes to cognitive impairment. Excessive oxidative stress exacerbates ferroptosis, collectively leading to declines in global cognition and memory, as confirmed by abundant previous studies.^44^, ^45^ After adjusting for age and[Fig alz70196-fig-0004] sex, iron deposition in the basal ganglia did not show a significant association with cSVD imaging markers, as reported in previous studies.^5^, ^46^ This inconsistency might be attributed to differences in the study populations and the definition of deep gray matter ROIs. Another finding was that increased susceptibility values in specific regions were associated with poorer performance in global cognition, executive function, and memory. Our findings are consistent with previous QSM studies based on hereditary cSVD, in which the involvement of the basal ganglia–thalamic circuit was thought to be associated with cognitive decline caused by deep gray matter iron deposition.^3^ Li et al. reported that in patients with cSVD, iron loss in WMH‐connected frontal and occipital regions, as well as cortical thinning, was associated with poorer processing speed.^47^ Although it did not reach statistical significance, we observed lower QSM values in the frontal lobe of the cSVD group. This pattern of susceptibility distribution, reflecting cortical demyelination, may also be linked to the cognitive impairment observed in cSVD patients in our study. Additionally, the combined effect of iron deposition and cSVD on cognitive decline merits further investigation, which we will discuss later.

Nevertheless, the detailed mechanism behind the area‐selective iron concentration in deep gray matter is still unclear. The imaging transcriptomics analysis used in our study could help explain the spatial pattern of iron deposition derived from the VBA. One of the major findings of our study is that synapse‐related gene sets and neuron‐specific genes were downregulated in regions that revealed higher QSM values in cSVD patients. We speculate that disrupted global brain signaling and metabolic processes, which function as an integrated system involving synaptic activity, iron uptake, and mitochondrial function under physiological conditions, may contribute to localized iron deposition.^48^ A QSM study focused on iron change within striatal subregions in Gilles de la Tourette syndrome reported the abnormalities of dopaminergic glutamatergic, and gamma‐aminobutyric acid (GABA)ergic transmission as the possible pathological mechanism of iron deficiency.^9^ Similarly, as depicted in our analysis, the enrichment of various synapse‐related gene sets, while not pointing to specific neurotransmitter pathways, may also support the association between disrupted CNS interconnections and iron deposition. Inflammation is another factor contributing to iron deposition, particularly in the context of our study on cSVD patients. Urrutia et al. reported that increased iron concentrations were only detected in neurons and microglia for up to 18 hours after short‐term inflammation stimulation.^49^ In our study, however, a different trend was observed, with neuron‐related genes being downregulated in regions with higher susceptibility values. Moreover, the distribution of microglia did not show consistency with the pattern of iron deposition. A possible explanation for this inconsistency is that the present results revealed more of the source of the QSM signal as myelin itself is diamagnetic and shows low QSM value, rather than the influence of the specific pathological process primary or secondary to ischemia. Therefore, our findings do not rule out the role of inflammation in iron deposition.

Among our findings, the enrichment of the GO term “endothelium development” and endothelial cell–specific genes from three single‐cell studies stands out, which, to our knowledge, has not been reported in previous QSM studies. This leads us to speculate that the blood–brain barrier (BBB) function may also play a role in brain iron deposition in cSVD patients. As a critical regulator of metal homeostasis in the CNS, brain vascular endothelial cells (BVECs) uptake iron mainly from plasma through the transferrin‐TFR1 system located on the luminal side of endothelial cells. The low molecular weight complex is then dissociated, with ferric iron released into the cytoplasm through divalent metal transporter 1, and subsequently released into the extracellular compartment via ferroportin (Fpn) or vesicular export. Brain regions with higher expression of endothelial cell–related genes might imply accordingly more extravasation of intravascular transferrin (Tf) when BBB is damaged.^43^ Fpn, as the primary exporter of iron, is widely distributed in the BBB and synaptic vesicles, further supporting the potential link between synaptic activity and iron deposition detected in this study.^50^ Evidence from clinical research using multi‐modality MRI, including diffusion‐prepared pseudo‐continuous arterial spin labeling (DP‐pCASL) and QSM, also supports the association between brain iron homeostasis and BBB function.^51^, ^52^ Nevertheless, the absence of significant enrichment in GO annotations directly related to the BBB function suggests that this mechanistic explanation should be approached with caution. In addition to the endothelial cells, the enrichment of genes specific to oligodendrocytes was expected, as oligodendrocytes typically store a large amount of iron for myelination under normal conditions.^53^ Iron is contained in oligodendrocytes in the form of ferritin, which is one of the main paramagnetic substances resulting in high contrast in QSM images.^54^ Under pathological conditions, hypoxia‐induced increases in ferritin synthesis within oligodendrocytes might also contribute to the observed enrichment of oligodendrocytes.^55^

The selective iron accumulation in deep nuclei resembles the previously reported patterns associated with aging, cerebrovascular diseases, and neurodegenerative diseases.^56^, ^57^, ^58^ This also suggests shared micro‐mechanisms underlying iron deposition in the deep gray matter. Our data show that iron deposition in the putamen and caudate interacts with PVS volume in the basal ganglia in relation to global cognition. Considering that the PVS burden is recognized to be associated with BBB dysfunction and changes in the glymphatic system,^59^ and that evidence from randomized controlled trials (RCTs) regarding deferiprone, a drug targeting iron deposition, does not support the idea that simply reversing iron deposition can improve cognitive function in neurodegenerative diseases,^60^, ^61^ these findings support our hypothesis that iron deposition in cSVD patients may result from structural or functional disruptions in the brain, rather than being the primary driver of cognitive decline. Taken together, we suggest that the disruption of brain structural and functional integrity may underpin the microscopic mechanisms driving localized iron deposition, while various complex cSVD‐related processes, such as oxidative stress, inflammatory infiltration, and endothelial dysfunction, also play critical roles.^62^, ^63^

Several limitations need to be acknowledged in the present study. First, as we used AHBA expression data to link spatial gene expression with practical neuroimaging findings, it is worth noting that this approach can only capture traits that are generally present in the human brain. In other words, in this study, we suggest that the genes or gene sets highly correlated with differences in iron deposition across brain regions might reflect inherent regional characteristics that predispose certain areas to greater iron accumulation, as indicated by changes in magnetic susceptibility. However, this approach does not directly validate secondary changes caused by pathological processes in patients, such as BBB disruption or secondary inflammatory changes, which were previously discussed as potential contributing factors. Meanwhile, the heterogeneity in the demographic characteristics of the AHBA donors and our study subjects must be acknowledged as a limitation. Second, advanced MRI techniques that could directly evaluate the BBB integration or function, such as DP‐pCASL and dynamic contrast‐enhanced MRI (DCE‐MRI) recommended for use in cSVD,^64^, ^65^, ^66^ were lacking in our study, which prevented us from further verifying our hypothesis for iron deposition. Finally, although offering high‐resolution spatial distribution of magnetic susceptibility, the specific sources of QSM signals remain unclear due to the complex tissue composition and the corresponding magnetic susceptibility within a single voxel. This implies that explanations for potential mechanisms of iron deposition in cSVD patients should be approached with caution.

In conclusion, we investigated the iron deposition pattern in cSVD patients at a macroscopic scale using QSM and further explored its potential mechanisms at the cellular and subcellular levels indirectly using AHBA expression data. Significant iron deposition was observed in the bilateral putamen and caudate nucleus, and higher QSM values in these regions were related to poorer cognitive function in cSVD patients. Transcriptomic analysis suggests that multifactorial mechanisms may contribute to the region‐specific iron deposition. Longitudinal research with a larger cohort is warranted to confirm the association between brain iron deposition and cognitive function or to explore the pathological mechanisms underlying iron deposition.

## CONFLICT OF INTEREST STATEMENT

The authors declare no conflict of interest. Author disclosures are available in the supporting information.

## CONSENT STATEMENT

Informed consent was provided by all subjects enrolled in the study.

## Supporting information



Supporting Information

Supporting Information
