# Supplementary material for: Cognitive implications and associated transcriptomic signatures of distinct regional iron depositions in cerebral small vessel disease
Source: Alzheimers Dement. 2025 Apr 21;21(4):e70196. doi: 10.1002/alz.70196 (PMC12010275; doi:10.1002/alz.70196)
Supplement: Supplementary file 1 — Supporting Information [file ALZ-21-e70196-s001.docx]

Title: Cognitive Implications and Associated Transcriptomic Signatures of Distinct Regional Iron Depositions in Cerebral Small Vessel Disease

**Supplementary Materials**

1. **Regions with Lower Susceptibility Value in cSVD Group**

| **Anatomical Structures** | **Voxel Size** | **Peak TFCE Value** | ***P* Value (TFCE FWE)** | **Peak Voxel MNI** | | |
| --- | --- | --- | --- | --- | --- | --- |
|  |  |  |  | X | Y | Z |
| Brainstem | 143 | 518.16 | 0.016 | 2 | -38 | -40 |
| Brainstem | 472 | 504.71 | 0.020 | 4 | -22 | -19 |
| Amygdala_R | 16 | 446.47 | 0.044 | 20 | -15 | -12 |

**Supplementary Table 1.** Clusters with decreased QSM value in the cSVD group, derived from QSM voxel-based analysis.

1. **Voxel-Based Morphometry (VBM) Results**


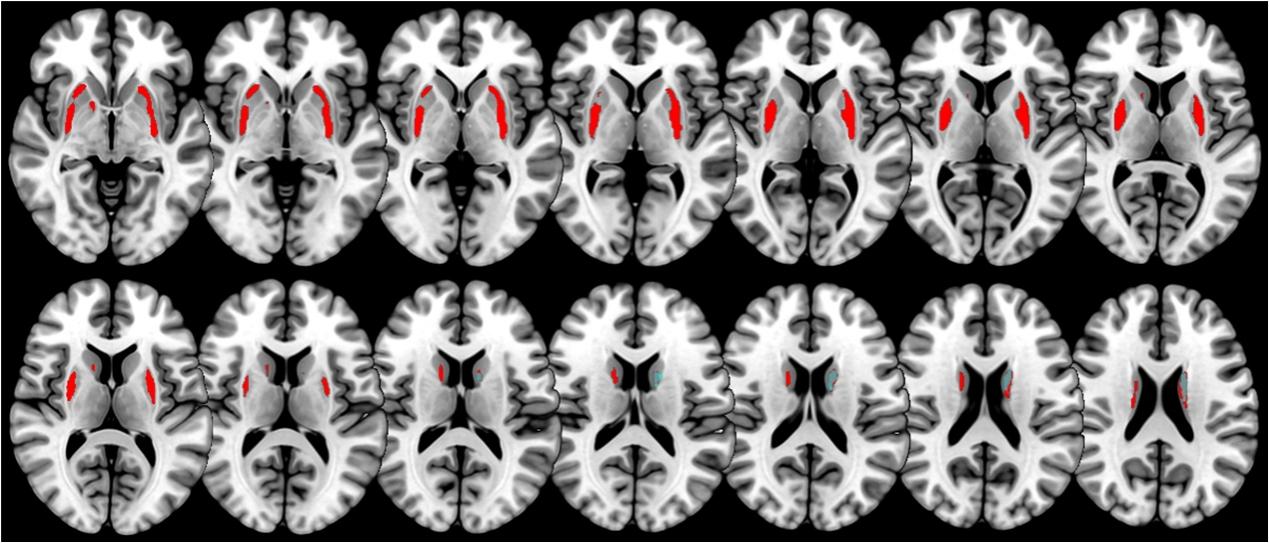


**Supplementary Figure 1.** Result of the VBM analysis. The red regions represent areas with increased susceptibility values according to the voxel-wise QSM analysis. A small cluster in the left caudate showed significantly higher grey matter volume in the cSVD group (in the lower row, blue overlay). No voxels survived in the VBM for the HC > cSVD contrast.

1. **Correlation Between Regional QSM Value and cSVD Imaging Markers**


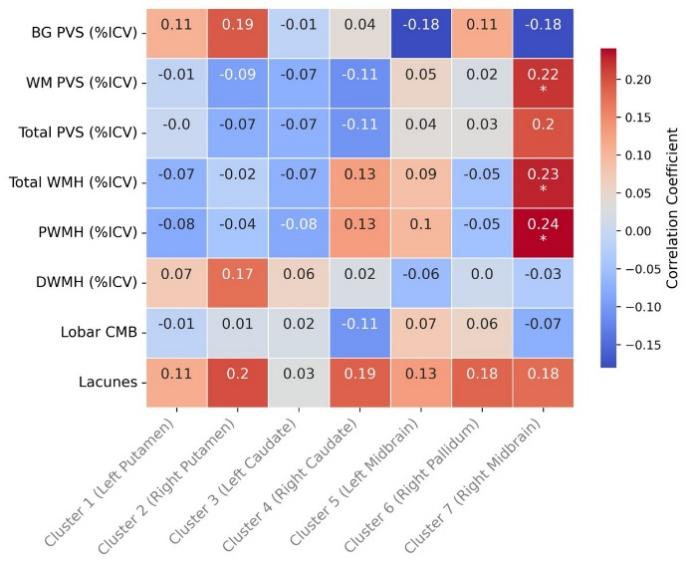


**Supplementary Figure 2.** Correlation between quantified cSVD imaging markers and susceptibility values in specific regions (clusters where cSVD > HC in VBA). The number at the top of each cell represents the partial correlation coefficient, adjusted for age and sex.

*: *P* < 0.05

1. **Correlation Between Regional QSM Value and Cognitive Function in cSVD Group (Model I)**

|  | Cluster 1 (Left Putamen) | | Cluster 2 (Right Putamen) | | Cluster 3 (Left Caudate) | | Cluster 4 (Right Caudate) | | Cluster 5 (Left Midbrain) | | Cluster 6 (Right Pallidum) | | Cluster 7 (Right Midbrain) | |
| --- | --- | --- | --- | --- | --- | --- | --- | --- | --- | --- | --- | --- | --- | --- |
|  | β (95% CI) | *P* Value | β (95% CI) | *P* Value | β (95% CI) | *P* Value | β (95% CI) | *P* Value | β (95% CI) | *P* Value | β (95% CI) | *P* Value | β (95% CI) | *P* Value |
| MoCA | -0.69  (-3.26 – 1.88) | 0.600 | -0.98  (-3.12 – 1.15) | 0.368 | -0.75  (-3.66 – 2.16) | 0.614 | **-4.97**  **(-7.86 – -2.08)** | **<0.001** | -0.38  (-1.75 – 0.99) | 0.589 | 0.24  (-0.96 – 1.43) | 0.696 | 0.02  (-1.40 – 1.44) | 0.980 |
| STT-A | 140.14  (-171.62 – 451.90) | 0.381 | **312.89**  **(64.66 – 561.11)** | **0.016** | 102.12  (-251.13 – 455.36) | 0.573 | 235.38  (-109.21 – 579.97) | 0.185 | 8.30  (-156.88 – 173.48) | 0.922 | 77.62  (-66.92 – 222.16) | 0.296 | 25.14  (-145.80 – 196.08) | 0.774 |
| STT-B | -309.70  (-1155.60 – 526.20) | 0.475 | -116.19  (-814.23 – 581.86) | 0.745 | -606.08  (-1555.323 – 343.17) | 0.215 | 309.85  (-631.81 – 1251.52) | 0.521 | -94.76  (-541.72 – 352.21) | 0.679 | -61.96  (-456.05 – 332.13) | 0.759 | -143.88  (-606.04 – 318.29) | 0.544 |
| Stroop-A_time_ | 50.72  (-60.10 – 161.53) | 0.372 | 47.54  (-43.53 – 138.60) | 0.309 | 74.30  (-50.45 – 199.05) | 0.247 | 104.44  (-17.27 – 226.15) | 0.097 | 22.07  (-36.45 – 80.59) | 0.462 | -12.94  (-64.61 – 38.74) | 0.625 | 35.36  (-24.92 – 95.65) | 0.254 |
| Stroop-B_time_ | 161.78  (-27.01 – 350.57) | 0.097 | **211.89**  **(60.94 – 362.84)** | **0.007** | 104.22  (-111.69 – 320.13) | 0.347 | 171.27  (-39.12 – 381.66) | 0.115 | 30.80  (-70.31 – 131.91) | 0.552 | -45.32  (-134.06 – 43.21) | 0.320 | 9.85  (-95.05 – 114.75) | 0.854 |
| Stroop-C_time_ | **421.77**  **(102.16 – 741.38)** | **0.012** | **375.18**  **(114.06 – 636.30)** | **0.006** | 335.09  (-33.84 – 704.01) | 0.079 | 340.42  (-22.39 – 703.23) | 0.070 | **208.94**  **(39.58 – 378.30)** | **0.018** | 65.89  (-88.22 – 220.01) | 0.405 | 79.69  (-101.32 – 260.70) | 0.391 |
| C-RAVLT-A1-5 | 0.22  (-1.83 – 2.26) | 0.836 | -0.16  (-1.87 – 1.55) | 0.851 | -1.38  (-3.72 – 0.97) | 0.250 | **-2.70**  **(-5.00 – -0.40)** | **0.022** | **-1.23**  **(-2.33 – -0.13)** | **0.029** | 0.03  (-0.94 – 1.00) | 0.955 | **-1.81**  **(-2.96** – **-0.67)** | **0.002** |
| C-RAVLT-A6 | 0.21  (-4.65 – 5.07) | 0.932 | -1.23  (-5.33 – 2.87) | 0.557 | -2.63  (-8.28 – 3.03) | 0.363 | -0.80  (-6.23 – 4.63) | 0.772 | -0.96  (-3.62 – 1.70) | 0.480 | 1.62  (-0.60 – 3.85) | 0.153 | -2.76  (-5.54 – 0.02) | 0.052 |
| C-RAVLT-A7 | -3.40  (-8.51 – 1.72) | 0.193 | -3.92  (-8.22 – 0.39) | 0.075 | -4.77  (-10.62 – 1.08) | 0.110 | **-6.44**  **(-12.13 – -0.75)** | **0.026** | -1.48  (-4.20 – 1.25) | 0.288 | 0.64  (-1.67 – 2.95) | 0.587 | -1.78  (-4.63 – 1.06) | 0.220 |

**Supplementary Table 2.** Correlation between QSM value and cognitive performance in cSVD patients. Regional QSM values were set as independent variables and cognitive performance was set as the dependent variable in the linear regression model. Age, sex, education level, and vascular risk factors were adjusted. MoCA, Montreal Cognitive Assessment-Beijing version; STT, Shape Trail Test; C-RAVLT, The Chinese Rey Auditory Verbal Learning Test; A1-5, the sum of five consecutive retrievals of the same words list; A6, retrieval of words without reading them again; A7, retrieval of the words without reading them again after a 30 minutes interval. Bold values are statistically significant.

1. **Correlation Between Regional QSM Value and Cognitive Function in cSVD Group (Model II)**

|  | Cluster 1 (Left Putamen) | | Cluster 2 (Right Putamen) | | Cluster 3 (Left Caudate) | | Cluster 4 (Right Caudate) | | Cluster 5 (Left Midbrain) | | Cluster 6 (Right Pallidum) | | Cluster 7 (Right Midbrain) | |
| --- | --- | --- | --- | --- | --- | --- | --- | --- | --- | --- | --- | --- | --- | --- |
|  | β (95% CI) | *P* Value | β (95% CI) | *P* Value | β (95% CI) | *P* Value | β (95% CI) | *P* Value | β (95% CI) | *P* Value | β (95% CI) | *P* Value | β (95% CI) | *P* Value |
| MoCA | -0.6  (-3.2 – 2) | 0.653 | -1.13  (-3.35 – 1.09) | 0.319 | -1.14  (-4.25 – 1.97) | 0.473 | **-4.9**  **(-8.02 – -1.77)** | **0.002** | -0.25  (-1.66 – 1.17) | 0.733 | 0.12  (-1.12 – 1.36) | 0.848 | 0.79  (-0.72 – 2.3) | 0.308 |
| STT-A | 176.17  (-158.82 – 511.15) | 0.306 | **369.06**  **(98.56 – 639.57)** | **0.009** | 111.61  (-285.06 – 508.28) | 0.583 | 154.33  (-240.6 – 549.25) | 0.446 | -59.2  (-239.69 – 121.29) | 0.522 | 31.73  (-128.36 – 191.82) | 0.699 | -60.2  (-253.15 – 132.76) | 0.543 |
| STT-B | -272.53  (-1138.57 – 593.52) | 0.539 | -81.71  (-812.12 – 648.7) | 0.827 | -690.62  (-1700.53 – 319.3) | 0.184 | 431.2  (-584.13 – 1446.52) | 0.408 | -91  (-556.25 – 374.25) | 0.703 | -36.65  (-448.89 – 375.59) | 0.862 | -216.24  (-711.43 – 278.96) | 0.395 |
| Stroop-A_time_ | 62.56  (-50.79 – 175.91) | 0.283 | 44.53  (-51.07 – 140.13) | 0.364 | 102.23  (-30.23 – 234.7) | 0.135 | 109.36  (-22.46 – 241.19) | 0.108 | 23.53  (-37.52 – 84.58) | 0.453 | -1.67  (-55.94 – 52.6) | 0.952 | 24.94  (-40.32 – 90.19) | 0.456 |
| Stroop-B_time_ | 183.29  (-14.22 – 380.8) | 0.073 | **254.16**  **(94.85 – 413.46)** | **0.003** | 167.93  (-66.86 – 402.72) | 0.165 | 230.41  (-0.87 – 461.68) | 0.055 | 45.23  (-62.65 – 153.12) | 0.414 | -24.43  (-120.23 – 71.37) | 0.619 | -12.59  (-128.41 – 103.23) | 0.832 |
| Stroop-C_time_ | **445.48**  **(106.38 – 784.58)** | **0.012** | **410.63**  **(128.03 – 693.23)** | **0.006** | 393.38  (-14.21 – 800.97) | 0.063 | **426.64**  **(21.93 – 831.35)** | **0.043** | **221.09**  **(37.99 – 404.19)** | **0.021** | 95.11  (-71.87 – 262.1) | 0.268 | 68.74  (-134 – 271.48) | 0.509 |
| C-RAVLT-A1-5 | -0.95  (-3.01 – 1.1) | 0.363 | -1.22  (-2.98 – 0.55) | 0.176 | **-2.53**  **(-5.00 – -0.05)** | **0.045** | **-4.6**  **(-7.07 – -2.13)** | **<0.001** | -1.12  (-2.25 – 0.01) | 0.051 | -0.05  (-1.05 – 0.94) | 0.919 | -1.01  (-2.21 – 0.18) | 0.096 |
| C-RAVLT-A6 | -0.82  (-5.66 – 4.02) | 0.740 | -2.64  (-6.86 – 1.58) | 0.220 | -2.18  (-8.15 – 3.78) | 0.473 | -4.35  (-10.2 – 1.5) | 0.145 | -0.51  (-3.23 – 2.2) | 0.711 | 1.77  (-0.49 – 4.04) | 0.125 | -0.86  (-3.74 – 2.02) | 0.558 |
| C-RAVLT-A7 | **-5.51**  **(-10.60 – -0.41)** | **0.034** | **-5.49**  **(-9.87 – -1.11)** | **0.014** | -5.72  (-11.83 – 0.39) | 0.066 | **-9.44**  **(-15.55 – -3.34)** | **0.002** | -0.9  (-3.67 – 1.86) | 0.521 | 0.69  (-1.59 – 2.97) | 0.555 | -0.01  (-2.91 – 2.9) | 0.997 |

**Supplementary Table 3.** Correlation between QSM value and cognitive performance in cSVD patients. Regional QSM values were set as independent variables and cognitive performance was set as the dependent variable in the linear regression model. Age, sex, education level, vascular risk factors, and cSVD burden were adjusted. MoCA, Montreal Cognitive Assessment-Beijing version; STT, Shape Trail Test; C-RAVLT, The Chinese Rey Auditory Verbal Learning Test; A1-5, the sum of five consecutive retrievals of the same words list; A6, retrieval of words without reading them again; A7, retrieval of the words without reading them again after a 30 minutes interval. Bold values are statistically significant.

1. **Results of the Interaction Analysis**

| **Outcome** | **Cluster 1 (Left Putamen)** | | **Cluster 2 (Right Putamen)** | | **Cluster 3 (Left Caudate)** | | **Cluster 4 (Right Caudate)** | |
| --- | --- | --- | --- | --- | --- | --- | --- | --- |
|  | β (95% CI) | *P* Value | β (95% CI) | *P* Value | β (95% CI) | *P* Value | β (95% CI) | *P* Value |
| **BG PVS Volume (%ICV)** | | | | | | | | |
| MoCA | 0.03 (-0.14 – 0.21) | 0.697 | -0.02 (-0.19 – 0.16)^*^ | 0.851 | -0.01 (-0.19 – 0.16) | 0.896 | -0.27 (-0.44 – -0.1) | 0.003 |
| STT-A | 0.1 (-0.09 – 0.29) | 0.292 | 0.24 (0.06 – 0.43) | 0.013 | 0.06 (-0.12 – 0.25) | 0.508 | 0.1 (-0.1 – 0.3) | 0.349 |
| STT-B | -0.06 (-0.24 – 0.13) | 0.561 | -0.04 (-0.24 – 0.15) | 0.668 | -0.08 (-0.26 – 0.1) | 0.399 | 0.08 (-0.12 – 0.28) | 0.448 |
| Stroop-A_time_ | 0.13 (-0.08 – 0.33) | 0.234 | 0.1 (-0.11 – 0.31) | 0.355 | 0.15 (-0.06 – 0.35) | 0.160 | 0.17 (-0.05 – 0.39) | 0.131 |
| Stroop-B_time_ | 0.15 (-0.06 – 0.36) | 0.169 | 0.27 (0.06 – 0.48) | 0.014 | 0.09 (-0.12 – 0.3) | 0.405 | 0.18 (-0.05 – 0.4) | 0.127 |
| Stroop-C_time_ | 0.3 (0.07 – 0.52) | 0.012 | 0.3 (0.07 – 0.53) | 0.012 | 0.22 (0 – 0.45) | 0.056 | 0.19 (-0.06 – 0.43) | 0.137 |
| C-RAVLT-A1-5 | 0.01 (-0.2 – 0.22) | 0.908 | 0.01 (-0.21 – 0.22) | 0.958 | -0.06 (-0.27 – 0.14) | 0.555 | -0.15 (-0.37 – 0.07) | 0.184 |
| C-RAVLT-A6 | -0.02 (-0.23 – 0.19) | 0.878 | -0.04 (-0.25 – 0.18) | 0.728 | -0.07 (-0.28 – 0.14) | 0.520 | -0.03 (-0.26 – 0.19) | 0.784 |
| C-RAVLT-A7 | -0.11 (-0.32 – 0.09) | 0.283 | -0.1 (-0.31 – 0.11) | 0.370 | -0.11 (-0.32 – 0.09) | 0.286 | -0.16 (-0.37 – 0.06) | 0.164 |
| **WM PVS Volume (%ICV)** | | | | | | | | |
| MoCA | -0.01 (-0.18 – 0.17) | 0.916 | -0.07 (-0.25 – 0.1) | 0.415 | -0.07 (-0.24 – 0.1) | 0.436 | -0.29 (-0.47 – -0.12) | 0.002 |
| STT-A | 0.07 (-0.11 – 0.26) | 0.443 | 0.2 (0.02 – 0.38) | 0.036 | 0.02 (-0.17 – 0.21) | 0.823 | 0.1 (-0.1 – 0.31) | 0.311 |
| STT-B | -0.08 (-0.27 – 0.11) | 0.413 | -0.02 (-0.21 – 0.17) | 0.820 | -0.13 (-0.32 – 0.05) | 0.172 | 0.06 (-0.13 – 0.26) | 0.522 |
| Stroop-A_time_ | 0.1 (-0.1 – 0.3) | 0.329 | 0.13 (-0.07 – 0.33) | 0.216 | 0.17 (-0.03 – 0.37) | 0.109 | 0.16 (-0.05 – 0.38) | 0.143 |
| Stroop-B_time_ | 0.18 (-0.02 – 0.39) | 0.085 | 0.33 (0.12 – 0.53) | 0.003 | 0.16 (-0.04 – 0.37) | 0.128 | 0.21 (-0.02 – 0.43) | 0.083 |
| Stroop-C_time_ | 0.28 (0.06 – 0.5) | 0.014 | 0.32 (0.1 – 0.54) | 0.006 | 0.23 (0.01 – 0.45) | 0.048 | 0.2 (-0.05 – 0.44) | 0.118 |
| C-RAVLT-A1-5 | -0.02 (-0.22 – 0.18) | 0.854 | -0.03 (-0.24 – 0.18) | 0.750 | -0.12 (-0.32 – 0.09) | 0.262 | -0.18 (-0.4 – 0.04) | 0.117 |
| C-RAVLT-A6 | -0.02 (-0.22 – 0.19) | 0.861 | -0.08 (-0.29 – 0.13) | 0.476 | -0.07 (-0.27 – 0.14) | 0.517 | -0.07 (-0.29 – 0.16) | 0.563 |
| C-RAVLT-A7 | -0.13 (-0.34 – 0.07) | 0.208 | -0.14 (-0.35 – 0.07) | 0.197 | -0.12 (-0.32 – 0.09) | 0.259 | -0.19 (-0.41 – 0.03) | 0.089 |
| **Total PVS Volume (%ICV)** | | | | | | | | |
| MoCA | -0.01 (-0.19 – 0.17) | 0.915 | -0.07 (-0.25 – 0.11) | 0.447 | -0.07 (-0.25 – 0.11) | 0.434 | -0.29 (-0.47 – -0.11) | 0.002 |
| STT-A | 0.07 (-0.11 – 0.26) | 0.449 | 0.2 (0.02 – 0.38) | 0.036 | 0.02 (-0.17 – 0.21) | 0.822 | 0.1 (-0.1 – 0.31) | 0.314 |
| STT-B | -0.08 (-0.27 – 0.11) | 0.408 | -0.02 (-0.21 – 0.17) | 0.824 | -0.13 (-0.31 – 0.06) | 0.185 | 0.07 (-0.13 – 0.26) | 0.515 |
| Stroop-A_time_ | 0.1 (-0.1 – 0.3) | 0.318 | 0.13 (-0.07 – 0.33) | 0.213 | 0.17 (-0.03 – 0.37) | 0.101 | 0.17 (-0.05 – 0.38) | 0.142 |
| Stroop-B_time_ | 0.18 (-0.02 – 0.39) | 0.082 | 0.33 (0.12 – 0.53) | 0.003 | 0.17 (-0.04 – 0.37) | 0.119 | 0.21 (-0.02 – 0.43) | 0.081 |
| Stroop-C_time_ | 0.28 (0.06 – 0.5) | 0.014 | 0.32 (0.1 – 0.54) | 0.007 | 0.23 (0.01 – 0.46) | 0.043 | 0.2 (-0.05 – 0.44) | 0.119 |
| C-RAVLT-A1-5 | -0.02 (-0.22 – 0.18) | 0.850 | -0.03 (-0.24 – 0.18) | 0.778 | -0.12 (-0.32 – 0.09) | 0.260 | -0.18 (-0.4 – 0.04) | 0.120 |
| C-RAVLT-A6 | -0.02 (-0.22 – 0.19) | 0.869 | -0.07 (-0.28 – 0.14) | 0.493 | -0.07 (-0.28 – 0.13) | 0.496 | -0.07 (-0.29 – 0.16) | 0.563 |
| C-RAVLT-A7 | -0.13 (-0.34 – 0.07) | 0.210 | -0.14 (-0.35 – 0.07) | 0.203 | -0.12 (-0.33 – 0.08) | 0.251 | -0.19 (-0.41 – 0.03) | 0.087 |
| **Total WMH Volume (%ICV)** | | | | | | | | |
| MoCA | -0.03 (-0.21 – 0.14) | 0.706 | -0.08 (-0.25 – 0.1) | 0.393 | -0.05 (-0.22 – 0.12) | 0.574 | -0.28 (-0.45 – -0.11) | 0.002 |
| STT-A | 0.1 (-0.09 – 0.29) | 0.311 | 0.25 (0.07 – 0.43) | 0.009 | 0.05 (-0.14 – 0.23) | 0.632 | 0.1 (-0.1 – 0.3) | 0.329 |
| STT-B | -0.07 (-0.26 – 0.12) | 0.485 | -0.03 (-0.22 – 0.16) | 0.733 | -0.13 (-0.31 – 0.06) | 0.190 | 0.06 (-0.14 – 0.26) | 0.572 |
| Stroop-A_time_ | 0.11 (-0.09 – 0.31) | 0.299 | 0.09 (-0.11 – 0.29) | 0.402 | 0.14 (-0.06 – 0.33) | 0.180 | 0.18 (-0.04 – 0.39) | 0.110 |
| Stroop-B_time_ | 0.19 (-0.03 – 0.4) | 0.089 | 0.31 (0.11 – 0.52) | 0.004 | 0.14 (-0.07 – 0.36) | 0.195 | 0.2 (-0.03 – 0.42) | 0.099 |
| Stroop-C_time_ | 0.31 (0.09 – 0.53) | 0.008 | 0.32 (0.1 – 0.55) | 0.006 | 0.18 (-0.04 – 0.41) | 0.117 | 0.23 (-0.02 – 0.47) | 0.077 |
| C-RAVLT-A1-5 | -0.07 (-0.27 – 0.13) | 0.518 | -0.08 (-0.28 – 0.12) | 0.447 | -0.08 (-0.28 – 0.11) | 0.401 | -0.25 (-0.46 – -0.04) | 0.024 |
| C-RAVLT-A6 | -0.04 (-0.25 – 0.18) | 0.740 | -0.08 (-0.29 – 0.13) | 0.448 | -0.02 (-0.23 – 0.19) | 0.821 | -0.05 (-0.28 – 0.17) | 0.645 |
| C-RAVLT-A7 | -0.18 (-0.38 – 0.03) | 0.095 | -0.18 (-0.39 – 0.02) | 0.083 | -0.12 (-0.32 – 0.08) | 0.256 | -0.21 (-0.43 – 0) | 0.059 |
| **PWMH Volume (%ICV)** | | | | | | | | |
| MoCA | -0.03 (-0.21 – 0.14) | 0.705 | -0.08 (-0.25 – 0.09) | 0.367 | -0.05 (-0.23 – 0.12) | 0.564 | -0.28 (-0.45 – -0.1) | 0.003 |
| STT-A | 0.1 (-0.09 – 0.29) | 0.319 | 0.25 (0.07 – 0.43) | 0.009 | 0.04 (-0.15 – 0.23) | 0.650 | 0.1 (-0.1 – 0.31) | 0.330 |
| STT-B | -0.07 (-0.26 – 0.12) | 0.482 | -0.03 (-0.22 – 0.16) | 0.753 | -0.12 (-0.31 – 0.06) | 0.201 | 0.06 (-0.15 – 0.26) | 0.594 |
| Stroop-A_time_ | 0.11 (-0.09 – 0.31) | 0.302 | 0.09 (-0.11 – 0.29) | 0.373 | 0.14 (-0.06 – 0.34) | 0.173 | 0.18 (-0.04 – 0.39) | 0.112 |
| Stroop-B_time_ | 0.19 (-0.03 – 0.4) | 0.091 | 0.32 (0.11 – 0.52) | 0.004 | 0.14 (-0.07 – 0.36) | 0.199 | 0.2 (-0.03 – 0.43) | 0.100 |
| Stroop-C_time_ | 0.31 (0.08 – 0.53) | 0.009 | 0.32 (0.1 – 0.55) | 0.006 | 0.19 (-0.04 – 0.42) | 0.118 | 0.22 (-0.02 – 0.47) | 0.079 |
| C-RAVLT-A1-5 | -0.06 (-0.27 – 0.14) | 0.541 | -0.08 (-0.28 – 0.12) | 0.448 | -0.08 (-0.28 – 0.12) | 0.412 | -0.25 (-0.46 – -0.04) | 0.024 |
| C-RAVLT-A6 | -0.04 (-0.25 – 0.18) | 0.746 | -0.08 (-0.3 – 0.13) | 0.438 | -0.02 (-0.23 – 0.19) | 0.846 | -0.05 (-0.27 – 0.17) | 0.663 |
| C-RAVLT-A7 | -0.17 (-0.38 – 0.03) | 0.097 | -0.18 (-0.39 – 0.02) | 0.082 | -0.12 (-0.32 – 0.09) | 0.260 | -0.21 (-0.43 – 0.01) | 0.062 |
| **DWMH Volume (%ICV)** | | | | | | | | |
| MoCA | -0.02 (-0.2 – 0.15) | 0.785 | -0.05 (-0.23 – 0.13) | 0.588 | -0.05 (-0.22 – 0.13) | 0.591 | -0.32 (-0.49 – -0.15) | 0.001 |
| STT-A | 0.08 (-0.1 – 0.27) | 0.384 | 0.21 (0.03 – 0.39) | 0.024 | 0.05 (-0.13 – 0.23) | 0.554 | 0.11 (-0.08 – 0.3) | 0.270 |
| STT-B | -0.07 (-0.26 – 0.12) | 0.450 | -0.06 (-0.25 – 0.13) | 0.555 | -0.14 (-0.32 – 0.05) | 0.147 | 0.06 (-0.14 – 0.25) | 0.562 |
| Stroop-A_time_ | 0.1 (-0.1 – 0.31) | 0.333 | 0.06 (-0.15 – 0.26) | 0.596 | 0.15 (-0.05 – 0.35) | 0.145 | 0.18 (-0.04 – 0.39) | 0.110 |
| Stroop-B_time_ | 0.18 (-0.03 – 0.39) | 0.101 | 0.28 (0.07 – 0.48) | 0.010 | 0.13 (-0.08 – 0.34) | 0.243 | 0.22 (0 – 0.45) | 0.053 |
| Stroop-C_time_ | 0.31 (0.08 – 0.53) | 0.009 | 0.33 (0.11 – 0.56) | 0.005 | 0.23 (0 – 0.45) | 0.050 | 0.22 (-0.02 – 0.47) | 0.080 |
| C-RAVLT-A1-5 | -0.06 (-0.26 – 0.14) | 0.551 | -0.06 (-0.26 – 0.15) | 0.590 | -0.14 (-0.33 – 0.05) | 0.148 | -0.24 (-0.44 – -0.03) | 0.027 |
| C-RAVLT-A6 | -0.03 (-0.25 – 0.18) | 0.766 | -0.08 (-0.3 – 0.13) | 0.453 | -0.04 (-0.25 – 0.17) | 0.692 | -0.08 (-0.31 – 0.14) | 0.478 |
| C-RAVLT-A7 | -0.16 (-0.37 – 0.04) | 0.119 | -0.18 (-0.39 – 0.02) | 0.088 | -0.14 (-0.34 – 0.06) | 0.188 | -0.2 (-0.41 – 0.01) | 0.071 |
| **Lacunes** | | | | | | | | |
| MoCA | 0.07 (-0.2 – 0.33) | 0.617 | -0.08 (-0.37 – 0.21) | 0.601 | 0.04 (-0.21 – 0.3) | 0.748 | -0.16 (-0.45 – 0.12) | 0.268 |
| STT-A | 0.12 (-0.15 – 0.4) | 0.394 | 0.28 (0 – 0.56) | 0.060 | 0.15 (-0.12 – 0.42) | 0.272 | 0.32 (0 – 0.65) | 0.057 |
| STT-B | -0.08 (-0.36 – 0.2) | 0.578 | -0.09 (-0.4 – 0.22) | 0.578 | -0.12 (-0.39 – 0.15) | 0.393 | 0.12 (-0.22 – 0.46) | 0.497 |
| Stroop-A_time_ | 0.19 (-0.09 – 0.47) | 0.183 | 0.17 (-0.14 – 0.48) | 0.293 | 0.18 (-0.09 – 0.45) | 0.208 | 0.25 (-0.08 – 0.58) | 0.138 |
| Stroop-B_time_ | 0.08 (-0.22 – 0.39) | 0.587 | 0.29 (-0.04 – 0.62) | 0.089 | 0.02 (-0.27 – 0.32) | 0.877 | 0.19 (-0.17 – 0.55) | 0.297 |
| Stroop-C_time_ | 0.29 (-0.03 – 0.62) | 0.084 | 0.46 (0.11 – 0.81) | 0.012 | 0.25 (-0.06 – 0.57) | 0.126 | 0.26 (-0.14 – 0.66) | 0.209 |
| C-RAVLT-A1-5 | -0.18 (-0.46 – 0.1) | 0.205 | -0.27 (-0.58 – 0.03) | 0.085 | -0.22 (-0.49 – 0.04) | 0.110 | -0.4 (-0.72 – -0.07) | 0.020 |
| C-RAVLT-A6 | -0.15 (-0.46 – 0.15) | 0.323 | -0.29 (-0.63 – 0.04) | 0.092 | -0.14 (-0.43 – 0.16) | 0.375 | -0.24 (-0.61 – 0.13) | 0.205 |
| C-RAVLT-A7 | -0.24 (-0.52 – 0.04) | 0.106 | -0.4 (-0.7 – -0.09) | 0.014 | -0.17 (-0.45 – 0.11) | 0.246 | -0.33 (-0.67 – 0.01) | 0.063 |
| **Lobar CMB** | | | | | | | | |
| MoCA | 0.01 (-0.17 – 0.2) | 0.887 | 0.01 (-0.18 – 0.2) | 0.917 | -0.01 (-0.21 – 0.19) | 0.929 | -0.25 (-0.44 – -0.05) | 0.015 |
| STT-A | 0.1 (-0.09 – 0.29) | 0.318 | 0.23 (0.03 – 0.42) | 0.027 | 0.11 (-0.1 – 0.31) | 0.317 | 0.12 (-0.09 – 0.34) | 0.270 |
| STT-B | -0.06 (-0.26 – 0.13) | 0.542 | -0.1 (-0.3 – 0.1) | 0.337 | -0.09 (-0.3 – 0.12) | 0.403 | 0.12 (-0.1 – 0.33) | 0.296 |
| Stroop-A_time_ | 0.12 (-0.1 – 0.33) | 0.289 | 0.1 (-0.13 – 0.32) | 0.397 | 0.2 (-0.03 – 0.43) | 0.086 | 0.2 (-0.04 – 0.44) | 0.103 |
| Stroop-B_time_ | 0.15 (-0.07 – 0.37) | 0.189 | 0.25 (0.03 – 0.48) | 0.027 | 0.13 (-0.11 – 0.37) | 0.281 | 0.18 (-0.07 – 0.43) | 0.162 |
| Stroop-C_time_ | 0.27 (0.04 – 0.5) | 0.027 | 0.26 (0.02 – 0.5) | 0.035 | 0.23 (-0.03 – 0.48) | 0.083 | 0.21 (-0.05 – 0.48) | 0.117 |
| C-RAVLT-A1-5 | 0 (-0.21 – 0.22) | 0.979 | 0.03 (-0.19 – 0.25) | 0.788 | -0.07 (-0.3 – 0.16) | 0.557 | -0.11 (-0.35 – 0.12) | 0.358 |
| C-RAVLT-A6 | -0.04 (-0.26 – 0.18) | 0.725 | -0.06 (-0.28 – 0.17) | 0.615 | -0.09 (-0.33 – 0.14) | 0.433 | 0 (-0.24 – 0.24) | 0.997 |
| C-RAVLT-A7 | -0.11 (-0.32 – 0.1) | 0.300 | -0.09 (-0.3 – 0.13) | 0.439 | -0.09 (-0.32 – 0.14) | 0.437 | -0.09 (-0.32 – 0.14) | 0.447 |

**Supplementary Table 3.** Correlation between QSM values and MoCA with the interaction term QSM value * cSVD neuroimaging metrics. The table presents the main effect results from regression analyses where cognitive function is the dependent variable, QSM value is the independent variable, and the interaction term is included. Each subtable includes one cSVD marker added to the interaction term, along with the results for the main effects (with QSM value as the independent variable). Except for the interaction between Cluster 2 QSM values and BG PVS volume, which survived multiple comparison corrections (*P* < 0.00625), the remaining interaction terms did not reach statistical significance.

1. **Allen Human Brain Atlas (AHBA) Gene Expression Data Processing**

First, microarray probes were reannotated using data provided by Arnatkeviciute et al. [1]; probes not matched to a valid Entrez ID were discarded. Next, probes were filtered based on their expression intensity relative to background noise [2], such that probes with intensity less than the background in ≥50.00% of samples across donors were discarded, yielding 31,569 probes. When multiple probes indexed the expression of the same gene, we selected and used the probe with the most consistent pattern of regional variation across donors (i.e., differential stability [3]), calculated with:

$$\Delta_{s}\left( p \right)= \frac{1}{\binom{N}{2}}\sum_{i=1}^{N-1} \sum_{j=i+1}^{N} \rho\left[ B_{i}\left( p \right),B_{j}(p) \right]$$

where $\rho$ is Spearman's rank correlation of the expression of a single probe, $p$, across regions in two donors $B_{i}$ and $B_{j}$, and $N$ is the total number of donors. Here, regions correspond to the structural designations provided in the ontology from the AHBA.

The MNI coordinates of tissue samples were updated to those generated via non-linear registration using the Advanced Normalization Tools (ANTs; <https://github.com/chrisfilo/alleninf>). Samples were assigned to brain regions in the provided atlas if their MNI coordinates were within 2 mm of a given parcel. To reduce the potential for misassignment, sample-to-region matching was constrained by hemisphere and gross structural divisions (i.e., cortex, subcortex/brainstem, and cerebellum, such that e.g., a sample in the left cortex could only be assigned to an atlas parcel in the left cortex [1]). If a brain region was not assigned a tissue sample based on the above procedure, every voxel in the region was mapped to the nearest tissue sample from the donor in order to generate a dense, interpolated expression map. The average of these expression values was taken across all voxels in the region, weighted by the distance between each voxel and the sample mapped to it, in order to obtain an estimate of the parcellated expression values for the missing region. All tissue samples not assigned to a brain region in the provided atlas were discarded.

Inter-subject variation was addressed by normalizing tissue sample expression values across genes using a robust sigmoid function [4]:

$$x_{norm}=\frac{1}{1+exp(-\frac{(x-\left\langle x \right\rangle)}{{IQR}_{x}})}$$

where $\left\langle x \right\rangle$ is the median and ${IQR}_{x}$ is the normalized interquartile range of the expression of a single tissue sample across genes. Normalized expression values were then rescaled to the unit interval:

$$x_{scaled}=\frac{x_{norm}-min(x_{norm})}{\max\left( x_{norm} \right)-min(x_{norm})}$$

Gene expression values were then normalized across tissue samples using an identical procedure. Samples assigned to the same brain region were averaged separately for each donor and then across donors, yielding a regional expression matrix with 83 rows, corresponding to brain regions, and 15,633 columns, corresponding to the retained genes.

1. **Cell-type Specific Gene Sets Derived from Specificity Index Analysis**

|  | Endothelial Cells | Neuron | OPC | Oligodendrocytes | Astrocytes | Microglia |
| --- | --- | --- | --- | --- | --- | --- |
| **GSE52564** | ABCB1  ABCC6  ACVRL1  ADAMTSL2  ADCY4  ADH1A  ADH1C  ADH1B  ANGPT2  APCDD1  APLNR  APOLD1  ARHGAP29  ARHGEF5  ASB4  C16ORF89  BCL6B  C1ORF115  CA4  CD34  CD40  CD93  CDC42EP3  CDH5  CDKN2B  CGNL1  CLDN5  CLEC14A  CLIC5  COL4A2  CSRP2  CTSW  CYYR1  DLL4  EGFL7  EGFL8  EMCN  ERG  ESAM  EXOC3L1  FAM124B  FGD5  FGFBP1  FLT1  FLT4  FN1  FOXF2  FOXQ1  FZD6  GIMAP4  … | ABCC8  ABLIM3  ACHE  AJAP1  ANKRD35  ATP1A3  VWA5A  BARHL2  BCL11A  BCL11B  BHLHE22  BMP5  CACNA1B  CACNA2D1  CACNA2D2  CACNA2D3  CALB2  CA10  CCBE1  CD274  CDH4  CDH8  CELF4  CELF5  CELF6  CELSR3  CHGB  CHODL  CLSTN2  CNR1  CNTNAP4  COL25A1  CPNE4  CPNE5  CPNE7  CRABP1  CRH  CRHBP  CRMP1  DACT1  DCX  DISP2  DLX1  DLX2  DLX5  DLX6  DMRT3  DPYSL5  DRD2  DYNC1I1  … | C1QL1  C1QL2  CDO1  CHRNA4  COL1A1  COL3A1  CSPG4  DCN  DLL3  FAM180A  GRIA3  GSX1  LHFPL3  LINGO3  LNX1  LUM  MEGF11  NXPH1  PCDH15  PDGFRA  PNLIP  PRKG2  SHC4  SLC22A6  SLITRK1  SSTR1  STK32A  SULF1 | ASPA  GJB1  GSN  HAPLN2  ITGB4  KLK6  MAL  MBP  MOBP  MOG  OPALIN  PLEKHB1  PPP1R14A | A2M  ALDH1L1  AQP4  AQP9  ATP13A4  BMPR1B  CBSL  CBS  CCDC80  CHRDL1  CTH  CYBRD1  DIO2  EGFR  ELOVL2  ENTPD2  EPHX2  F3  FABP7  FAM181A  FGFR3  FMO1  FZD10  GCNT4  GDPD2  GLI1  GLI2  GLI3  GRHL1  GRM3  HES5  HGF  ITGA7  ITIH3  KCNN3  LGR6  MAMDC2  NAT8  NAT8B  NCAN  NWD1  OTX1  P4HA3  PAMR1  PAQR6  PHKG1  PLA2G3  PLCD4  PPP1R3C  PTX3  … | ABCC3  ADORA3  BCL2A1  BLNK  C1QA  C1QB  C1QC  C3AR1  C5AR1  CCL8  CCL1  CCL3L3  CCL3L1  CCL18  CCL3  CCL4L2  CCL4  CCL7  CCR1  CCR5  CCR7  CCRL2  CD14  CD300A  CD300LD  CD37  CD83  CLEC10A  CRYBB1  CSF1R  CSF3R  FCGR1A  FCGR1B  FCRL2  FERMT3  GDF15  GNA15  GPR157  GPR183  HCK  HK3  IL10RA  IL1A  IL21R  IRF5  IRF8  ITGB2  KCNK6  LAG3  LAPTM5  ... |
| **GSE67835** | GJA4  TIE1  VCAM1  VANGL1  ALX3  UBL4B  TBX15  ZNF648  SELE  MIR488  CDA  ID3  CD34  MFAP2  CASQ2  CD52  TINAGL1  ECM1  LIN9  DYRK3  S100A4  S100A2  MIR3115  FMO2  PEAR1  PALMD  RHCE  BTBD19  AQP10  FAM72B  RUNX3  FMOD  OSR1  CYP1B1  MATN3  TCF7L1  EDAR  FAM124B  SGPP2  TWIST2  TFPI  COL3A1  KLHL30  SNORA75  SNORD20  KRTCAP3  IL1RL1  LINC00487  SNORA70F  SULT1C2  … | CELA2A  PTPRU  PAX7  TMEM61  L1TD1  GPR88  LMX1A  DPT  FMO1  LINC00184  LAMB3  PDC  MIR942  MIR921  PIN1P1  MIR3123  PMF1-BGLAP  MIR4684  SCARNA1  MIR3675  HSD3B1  HSD3BP4  POU5F1P4  KNCN  OR14I1  TMEM52  MIR320B2  HORMAD1  MIR4666A  RCAN3AS  MIR4677  FAM41C  SH2D5  LEFTY1  MIR765  DNASE2B  THEM5  SNORA62  OPN3  INSL5  LCE5A  S100A3  SPRR2G  MIR4258  DCST1  ADAM15  GLIS1  KISS1  TNN  PTGER3  … | PEX10  NEGR1-IT1  MAEL  SNORD45C  WDR64  S100A14  ELF3  MIR1295A  FMO6P  RGS13  UTS2  VWA3B  RNF103-CHMP3  LY75  SPC25  DAPL1  STON1  B3GALT1  C3ORF20  SPATA16  IGF2BP2  PCOLCE2  RARRES1  PARP15  CCR8  TGM4  LINC00575  HSD17B13  FLJ38576  TIGD4  GDNF  IL4  MIR4803  MIR146A  PCDHB15  SPINK6  HAVCR1  TREM1  MIR3925  MIR548B  PNLDC1  MIR3692  C6ORF58  LAMB4  MIR3609  ZC3HAV1L  IGFBP1  KIAA0087  MIR188  ZNF157  … | LDLRAP1  ARHGEF19  DMRTA2  SLC45A3  MIR761  MIR3121  MIR1278  TMCO2  GJC2  CHI3L2  CCDC17  MSH4  CDC20  HAPLN2  RNU11  GOLT1A  MIR3605  FAM177B  MDS2  CLCA4  MATN1  MIR181B1  MIR181A1  CA14  OR2L13  OR2L1P  GNRHR2  TMEM182  BOK  MIR933  TMEM198  MIR4436A  HOXD1  MIR4783  MIR558  LINC00486  MIR4434  DOK1  CKAP2L  GHSR  MIR4790  MIR16-2  GPR62  MIR4271  TNNC1  ANP32C  MIR4276  MIR367  MIR302D  MIR302A  … | HES5  FAM110D  PLA2G5  GJB5  C1ORF87  RPE65  LPAR3  SNRPD2P2  ANKRD45  MIR34A  MIR2682  EFNA4  RAB25  BGLAP  PDZK1IP1  ACOT11  IGFN1  UOX  OR2L2  KLHDC7A  SYPL2  SNORA44  S100A1  HRNR  OR2B11  MIR215  MIR194-1  CHI3L1  AKR7A3  SPOCD1  ZBTB37  MIR4794  SLC25A34  ATP6V1G3  PDZK1P1  NTSR2  SFXN5  OTX1  TBR1  MIR153-1  TUBA3E  INHA  SNORD70  MYADML  CYS1  ATP6V1B1  SP5  MIR4429  PFN4  ILDR1  … | PTAFR  DLGAP3  BATF3  FRRS1  PIGR  MIR4257  KIF17  SNORD45A  SNORD45B  HSPB7  TNFSF18  C1QA  C1QC  C1QB  S100A8  PDZK1  CSF3R  FCGR3A  GSTM1  CYTIP  LINC00309  SNORD82  MIR2467  IL1RL2  TFCP2L1  MIR26B  DHRS9  OXER1  CTLA4  IL1RN  ARHGAP25  IL1A  FOXD4L1  CDKL4  MIR2355  RTP4  TIGIT  CCR5  XIRP1  TTLL3  DAPP1  MIR548I2  SH3TC1  TLR1  HPGDS  PTGER4  LTC4S  MIR3660  RELL2  CD14  … |
| **GSE73721** | RAMP2  RPL21  RPS10  ANXA3  APOLD1  ATP10A  BTNL9  CAV1  CD34  CDH5  CLDN5  ECSCR  EDN3  ELTD1  EMCN  ERG  ESAM  GNG11  GPR116  HIGD1B  IFITM1  ITIH5  LEF1  LY6E  MECOM  MYCT1  MYL9  NOSTRIN  OMD  PALMD  PODXL  RERGL  RNU11  SDPR  SLC38A5  SLC6A12  ST6GALNAC1  TGM2  TIE1  TM4SF1  TMEM204  VWF | CALB1  CALB2  CCK  CDH9  CNR1  DLX1  ELAVL2  ENTPD3  FSTL5  GABRA1  GABRB2  GABRG2  GAD1  GAD2  GRIN2A  GRIN3A  INA  KCNC2  KCNQ5  KIT  NELL2  NXPH2  OPRK1  PCDHGC4  PCP4L1  PENK  PTHLH  RAB3C  RELN  SCG2  ST8SIA3  STMN2  SYNPR  SYT1  SYT13  SYT4  TAC1  VIP  ZMAT4 | NA | FOLH1  HSD11B1  ANLN  CLDN11  CNDP1  ENPP2  ERMN  GJB1  GPR37  HHIP  KLK6  MOBP  MOG  OPALIN  PLP1  S1PR5  TMEM125 | AGT  AGXT2L1  BMPR1B  F3  FGFR3  GJA1  GJB6  PAMR1  RGS20  SDC4  SLC14A1  SLC39A12  GPR98 | NA |

**Supplementary Table 5.** Cell-type specific gene sets derived from the specificity index (SI) analysis. Only the first 50 genes are displayed if the gene sets contain more than 50 genes. The order of genes does not represent their significant level in the SI analysis. OPC and microglia-specific gene sets are shown as not available (NA) because GSE73721 does not contain RNA-seq data for these two cell types. The full version of gene sets with codes used for processing can be found at <https://github.com/LuuuXG/Brain-Imaging-Transcriptomics-Scripts>. OPC, Oligodendrocyte Precursor Cells.

1. **Validation of Partial Least Squares Regression (PLSR) by Linear Regression**


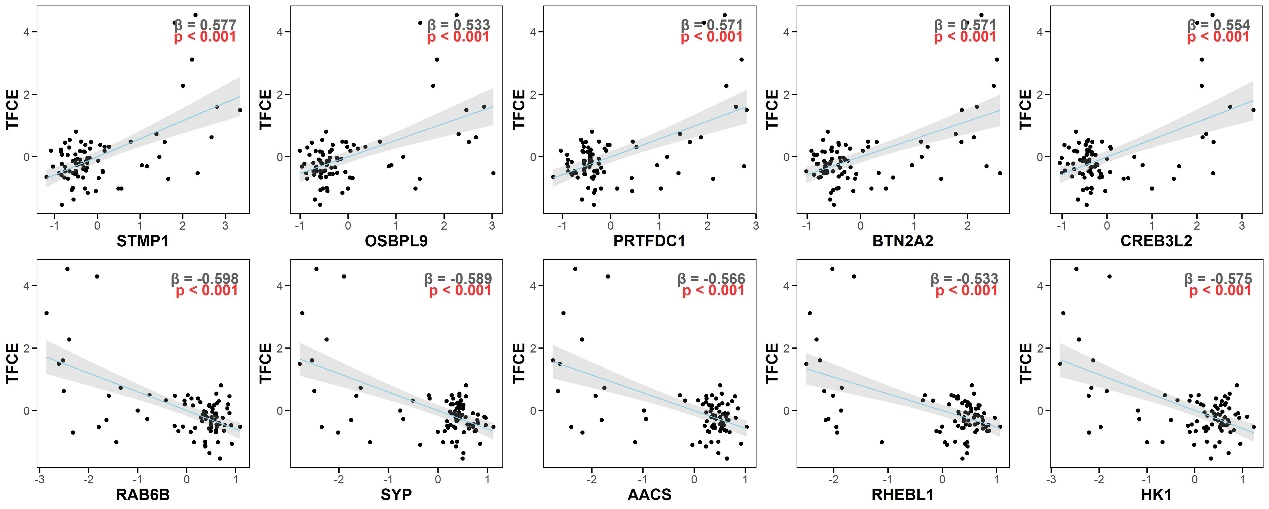


**Supplementary Figure 3.** Correlation between gene expression and TFCE values validated using linear regression with 1000 permutations. The top five genes ranked by PLSR Component 1 (shown in the first row) exhibit a positive correlation with TFCE values, while the bottom five genes (shown in the second row) exhibit a negative correlation with TFCE values.

1. **Sensitivity Analysis for Imaging Transcriptomics (Part I)**


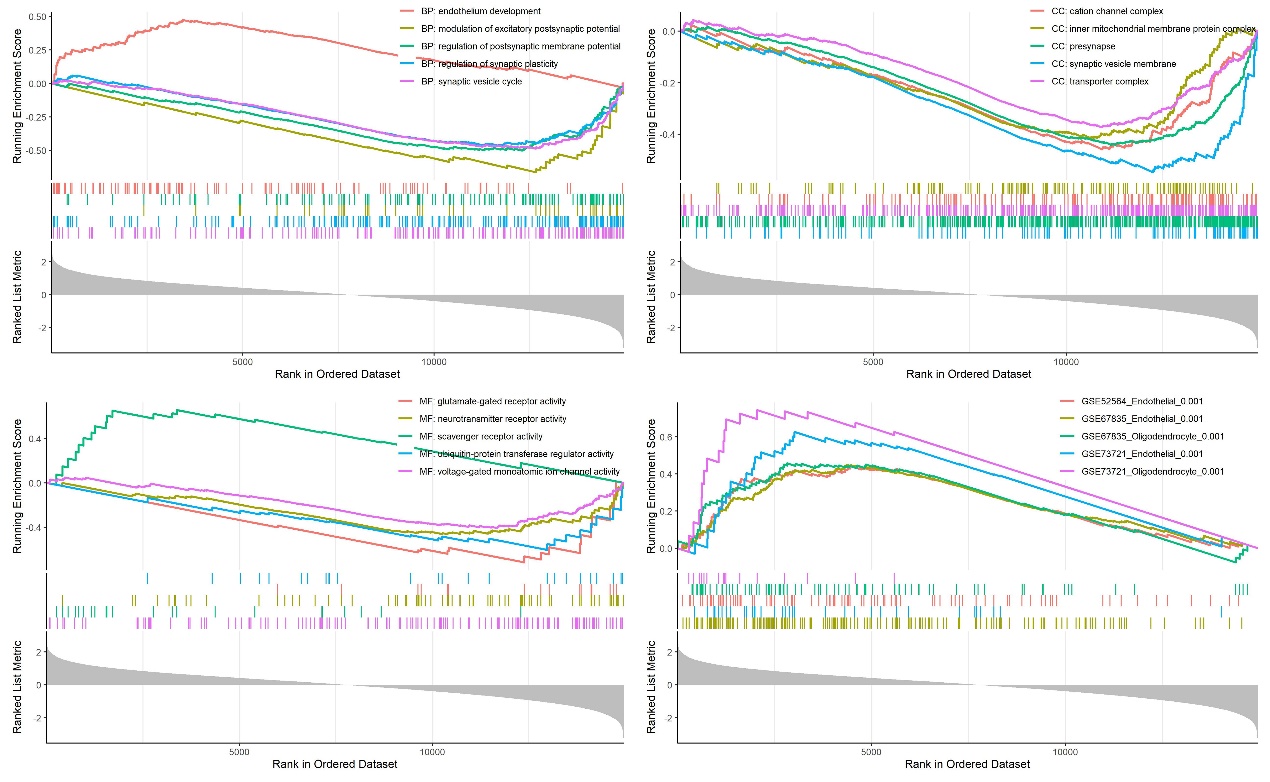


**Supplementary Figure 4.** Results of the enrichment analysis using the automated anatomical labeling (AAL) atlas with 90 cerebral regions.

1. **Sensitivity Analysis for Imaging Transcriptomics (Part II)**

The second part of the sensitivity analysis involved repeating PLS regression and GSEA on the BrainSpan transcriptomic dataset, following the methodology outlined by Buch et al. [5] Specifically, the raw RNA-Seq data in RPKM (reads per kilobase per million) were directly downloaded from the BrainSpan dataset ([https://www.brainspan.org](https://www.brainspan.org/static/home)). Participants under the age of 5 were excluded due to significant differences in brain tissue anatomy, leaving 13 donors (aged 8-40 years, 6 females). These donors represented 16 brain regions defined by BrainSpan. The gene expression values were averaged across the 16 brain regions for each donor. Since BrainSpan did not provide MNI space coordinates for the samples, these brain regions were manually mapped to the areas in the DK atlas (e.g., the caudate, putamen, and accumbens areas in the DK atlas were mapped to the striatum region in BrainSpan). In the end, the 16 BrainSpan regions were assigned to 44/83 brain regions in the DK atlas (with 22 regions assigned to each hemisphere). The subsequent analysis steps followed the same procedures as the main analysis.


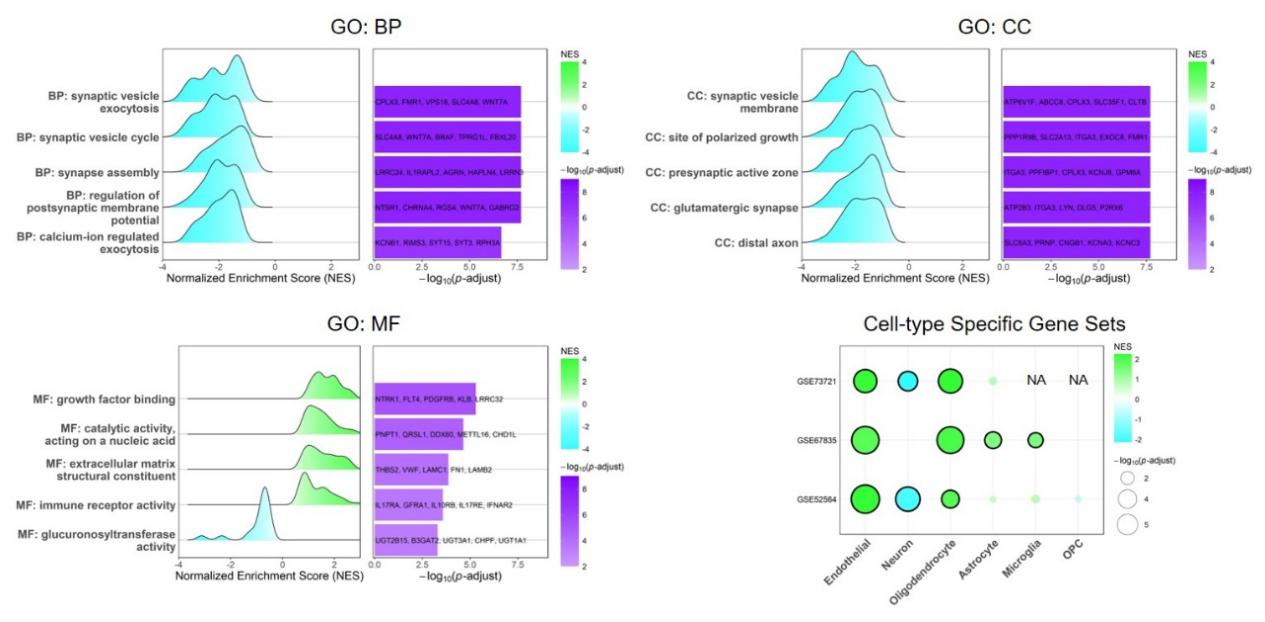


**Supplementary Figure 5.** Enriched gene sets and their NES, log-transformed P value from GSEA. For GO terms, the top five significant genesets of BP, CC, and MF were demonstrated with the first five core enrichment gene symbols. Results of cell-type specific gene sets were displayed as the bubble plot. Genes specific to neurons from GSE67835 did not show enrichment results due to insufficient overlap with the genes identified in BrainSpan.

BP, Biological Process; CC, Cellular Component; MF, Molecular Function.

**Reference**

[1] Arnatkeviciute A, Fulcher BD, Fornito A. A practical guide to linking brain-wide gene expression and neuroimaging data. Neuroimage. 2019;189:353-67.

[2] Quackenbush J. Microarray data normalization and transformation. Nat Genet. 2002;32 Suppl:496-501.

[3] Hawrylycz M, Miller JA, Menon V, Feng D, Dolbeare T, Guillozet-Bongaarts AL, et al. Canonical genetic signatures of the adult human brain. Nat Neurosci. 2015;18:1832-44.

[4] Fulcher BD, Little MA, Jones NS. Highly comparative time-series analysis: the empirical structure of time series and their methods. J R Soc Interface. 2013;10:20130048.

[5] Buch AM, Vertes PE, Seidlitz J, Kim SH, Grosenick L, Liston C. Molecular and network-level mechanisms explaining individual differences in autism spectrum disorder. Nat Neurosci. 2023;26:650-63.
